# Where genotype is not predictive of phenotype: towards an understanding of the molecular basis of reduced penetrance in human inherited disease

**DOI:** 10.1007/s00439-013-1331-2

**Published:** 2013-07-03

**Authors:** David N. Cooper, Michael Krawczak, Constantin Polychronakos, Chris Tyler-Smith, Hildegard Kehrer-Sawatzki

**Affiliations:** 1 Institute of Medical Genetics, School of Medicine, Cardiff University, Heath Park, Cardiff, CF14 4XN UK; 2 Institute of Medical Informatics and Statistics, Christian-Albrechts University, 24105 Kiel, Germany; 3 Department of Pediatrics/Human Genetics, McGill University Health Center, Montreal, Canada; 4 The Wellcome Trust Sanger Institute, Wellcome Trust Genome Campus, Hinxton, Cambridge, CB10 1SA UK; 5 Institute of Human Genetics, University of Ulm, Albert-Einstein-Allee 11, 89081 Ulm, Germany

## Abstract

Some individuals with a particular disease-causing mutation or genotype fail to express most if not all features of the disease in question, a phenomenon that is known as ‘reduced (or incomplete) penetrance’. Reduced penetrance is not uncommon; indeed, there are many known examples of ‘disease-causing mutations’ that fail to cause disease in at least a proportion of the individuals who carry them. Reduced penetrance may therefore explain not only why genetic diseases are occasionally transmitted through unaffected parents, but also why healthy individuals can harbour quite large numbers of potentially disadvantageous variants in their genomes without suffering any obvious ill effects. Reduced penetrance can be a function of the specific mutation(s) involved or of allele dosage. It may also result from differential allelic expression, copy number variation or the modulating influence of additional genetic variants in *cis* or in *trans*. The penetrance of some pathogenic genotypes is known to be age- and/or sex-dependent. Variable penetrance may also reflect the action of unlinked modifier genes, epigenetic changes or environmental factors. At least in some cases, complete penetrance appears to require the presence of one or more genetic variants at other loci. In this review, we summarize the evidence for reduced penetrance being a widespread phenomenon in human genetics and explore some of the molecular mechanisms that may help to explain this enigmatic characteristic of human inherited disease.

## Introduction

One old conundrum in human genetics is that not everyone with a given pathological mutation (or mutations) will eventually develop the disease in question. The proportion of those individuals harbouring a particular pathogenic mutation or genotype who exhibit clinical signs of the associated disorder within a specific and clearly defined time period is termed the *penetrance* of that disorder. If this proportion equals 100 %, the disease and/or disease genotype(s) are said to show complete penetrance. If not, they are said to exhibit reduced (or incomplete) penetrance. Reduced penetrance is likely to be a consequence of the combination of a variety of different genetic and environmental factors. A classic example is phenylketonuria, where inactivating mutations in the *PAH* gene encoding the enzyme phenylalanine hydroxylase lead to severe intellectual disability in the context of a normal diet, whereas a life-long phenylalanine-restricted diet makes possible a relatively healthy life (Blau et al. 2010). Regrettably, few other examples of reduced penetrance are as simple, well understood or clinically manipulable as this one, and the precise mechanisms by which different factors give rise to reduced penetrance remain largely unknown (Zlotogora 2003).

In formal terms, penetrance measures the proportion of individuals in a given population with a specific disease-associated genotype who also express the corresponding disease phenotype. Large family/population studies have traditionally been considered necessary to measure penetrance, either for specific mutations/genotypes or as a disease average. ‘Cascade genetic screening’, whereby relatives of previously identified carriers are screened both for the mutation(s) in question and for the presence/absence of clinical symptoms, is one means to determine the degree of penetrance for a given genotype (Berge et al. 2008). As we discuss below, large-scale sequencing and genotyping studies of apparently healthy individuals from the general population provide a powerful new approach to understanding the penetrance of pathological mutations/genotypes. The outcomes of such studies should allow us to predict how likely it is that a given disease will manifest itself in an individual who carries a specific genotype.

Reduced penetrance is most obviously evident in disorders that follow an autosomal dominant mode of inheritance. In these instances, reduced penetrance is a characteristic of the underlying mutation, rather than a genotype. However, reduced penetrance can also occur in autosomal recessive disorders where one and the same mutation can have different phenotypic effects, depending at least in part upon the second disease allele present. Irrespective of the mode of inheritance, in most cases penetrance is likely to be a function of the specific mutation(s) involved. Thus, in some conditions normally characterized by an autosomal dominant mode of inheritance, two incompletely penetrant (or otherwise non-penetrant) alleles may act in recessive fashion while mimicking the normal dominant form of the disease (e.g. Grundy et al. 1991; Croxen et al. 2002; Kowalewski et al. 2007; Castaman et al. 2007; Rossetti et al. 2009; Vujic et al. 2010; Schaaf et al. 2011a). For a dominantly inherited condition, one consequence of reduced penetrance is that the clinical phenotype may not be evident in one generation, but can nevertheless still be transmitted (through an apparently unaffected parent) to subsequent generations where it again manifests itself; specimen examples of this from clinical practice include hereditary hyperekplexia (Kwok et al. 2001), cherubism (Preda et al. 2010), retinitis pigmentosa (Rio Frio et al. 2009), rhabdoid tumour predisposition syndrome (Ammerlaan et al. 2008), autism spectrum disorder (Fujita-Jimbo et al. 2012) and hypercholesterolaemia (Garcia–Garcia et al. 2011). For all the above reasons, reduced penetrance presents a major challenge to genetic counsellors attempting to interpret the medical history of a patient’s family to quantify the disease risk to the patient’s offspring (Emery 1986; Otto and Maestrelli 2000).

Reduced penetrance is not uncommon; indeed, there are many known examples of bona fide disease-causing variants or genotypes that fail to cause disease in at least a proportion of individuals who carry them (Zlotogora 2003; Waalen and Beutler 2009). By definition, penetrance refers to the black and white issue of whether the clinical phenotype associated with a certain genotype is present or not. We routinely distinguish it from variable expressivity which refers to the degree of variation of the clinical phenotype in those individuals with a particular genotype. Although, in principle, penetrance and expressivity are distinct terms with specific meanings (depending upon the way a given clinical phenotype is defined), in practice they are closely inter-related and likely to manifest via similar mechanisms. We also distinguish reduced penetrance from small effect size. Thus, most carriers of the risk alleles discovered by genome-wide association studies (GWAS) may never develop the disease in question; this is because these variants generally only make a small contribution to the multifactorial aetiology of the condition. To be able instead to say that the variant is non-penetrant in some individuals, we require the same variant in other individual(s) to make the crucial difference between the phenotype being manifested or not. In what follows, we shall focus specifically on the molecular mechanisms that could account for the phenomenon of reduced penetrance. This notwithstanding, the discussion of genetic variants that modulate the expressivity of a particular disease has sometimes also been deemed appropriate.

In this review, we present the evidence for reduced penetrance being a widespread phenomenon in human genetics, evidence that comes not only from a plethora of case studies of monogenic disorders but also more recently from the next generation sequencing of entire exomes or genomes of apparently normal healthy individuals from the general population. Here, we have attempted to explore the individual genetic components that contribute to the complexity of primarily monogenic disorders with reduced penetrance. This notwithstanding, the action of modifier genes is one of the mechanisms responsible for reduced penetrance, and one that has increasingly become recognized as blurring the distinction between monogenic conditions and complex disease (Nadeau 2003; Badano and Katsanis 2003; Sidransky 2006). Finally, we explore some of the molecular mechanisms which could account for the reduced penetrance of many human inherited disorders and provide evidence to support the view that, at least in some instances, complete penetrance requires the presence of one or more genetic variants at other loci.

## Deleterious and disease alleles in the general population

In the wake of the sequencing of multiple human genomes, it has become apparent that healthy individuals can harbour quite large numbers of potentially disadvantageous variants without suffering any obvious ill effects (The 1000 Genomes Project Consortium 2010; MacArthur et al. 2012; Xue et al. 2012; Shen et al. 2013a). The underlying reasons are likely to be many and varied: thus, the variants may damage the protein in question but the intact protein may not be necessary for the health of the carrier; individuals may be asymptomatic carriers of single-mutant alleles that could, in homozygosity or compound heterozygosity, cause recessive disease; the mutation may be dominant but the clinical phenotype might only be mild and classed as lying within the range of normal healthy variation; the disorder might be late in onset with expression being age- or sex-dependent; or the disorder may require additional genetic and/or environmental factors for it to manifest clinically.

Assessing the magnitude of the ‘genetic burden’ imposed on the general population by the presence of deleterious alleles has been a key aim of medical and population genetics for many decades. With the advent of large-scale sequencing technologies, it has become possible to estimate the number of amino acid substitutions in the human exome that would be predicted in silico to be damaging (Kryukov et al. 2007; Lohmueller et al. 2008; Boyko et al. 2008; Goode et al. 2010). However, personal genome sequences have not only provided estimates of the number of disease variants carried by each subject (Asan et al. 2011), but have also given us a glimpse of the likely complexity of the functional interpretation of such data (Ashley et al. 2010; Strom and Gorin 2013). More recently, Tennessen et al. (2012) suggested that 2.3 % of the 13,595 single nucleotide variants carried by the average person impact upon protein function, involving ~313 genes per human genome. Adopting an alternative approach, Bell et al. (2011) surveyed 437 genes known to be related to a recessive Mendelian disease and identified 2.8 mutations per individual (range 0–7). Taken together, these studies suggest that individuals typically carry hundreds of mildly disadvantageous variants and perhaps several tens of potentially severe disease alleles.

In a pilot study for the 1000 Genomes Project Consortium (2010), we reported the prevalence of disease alleles, defined by reference to the disease-causing mutations (DMs) listed in the Human Gene Mutation Database (HGMD; http://www.hgmd.org; Stenson et al. 2009), in population samples of African, European and East Asian origin. These numbers were surprisingly high: 57–80 disease alleles per individual in a sample of 179 participants. Moreover, further examination of these data showed that 191 disease alleles were present in the homozygous state in at least one individual, and hence were not simply present because their phenotypic/clinical effects had been masked by a normal allele. Although little phenotypic information other than sex, ethnicity, place of origin and relationship to other participants is available for the 1000 Genomes Project donors, the Project’s ethical framework requires that sample donors are non-vulnerable adults who are competent to consent to participation in the project and hence are likely to lack any obvious severe disease phenotype. Instead, we need to seek some other explanation for the high number of DMs present. One possibility is that the penetrance of disease alleles and genotypes could be much lower and more variable than previously realized. Conventionally, most studies of human inherited disease that have contributed mutation data to HGMD have sought to identify a disease genotype, given a clinical phenotype (Cooper et al. 2010). This is a very different scenario from identifying a phenotype given a (potentially disease associated) genotype. Indeed, the former strategy inevitably avoids the whole issue of penetrance because, by definition, it focuses exclusively upon those individuals in whom the mutation of interest has been penetrant. It follows that a given variant could be genuinely causative in a set of individuals manifesting a particular disease, yet may also be present in a set of healthy individuals who differ in any one of a number of ways to be discussed below.

Since the 1000 Genomes Project Consortium paper was published in 2010, two further reports have appeared that served to improve our knowledge of deleterious mutations in the genomes of apparently healthy individuals. The most readily recognized deleterious variants in the human genome are those that disrupt a protein-coding gene, either by leading to a loss of function (e.g. a nonsense or frameshift variant) or by altering an amino acid in the encoded protein (missense variants). The former category of mutation has been studied by MacArthur et al. (2012) who identified 1,285 putative loss-of-function variants (i.e. nonsense mutations, splice site-disrupting single nucleotide variants, micro-insertions/micro-deletions, etc.) in the genome sequences of 185 humans from the 1000 Genomes Project. From these data, they estimated that an average human genome typically contains ~100 genuine loss-of-function variants with ~20 genes having both copies inactivated. Following on from this study, Xue et al. (2012) focused on missense mutations, ascertaining the numbers of potentially deleterious missense variants in the genomes of apparently healthy individuals using low-coverage whole-genome sequence data from 179 individuals in the 1000 Genomes Pilot Project. Each individual was found to carry 281–515 missense substitutions predicted with a high degree of confidence to be damaging to the gene product, 40–85 of which were present in the homozygous state. Taken together, these studies suggest that a typical healthy individual has about 80 of their genes severely damaged or inactivated in both copies, further emphasizing the stark contrast between damage to gene and protein on the one hand, and damage to health on the other. The 1000 Genomes Project participants also carried 40–110 variants (3–24 homozygous) classified by HGMD as DMs. Whereas many of these DMs could conceivably represent disease attribution errors of some kind, between 0 and 8 DMs per individual (0–1 homozygous) were predicted to be highly damaging.

Among the missense DMs, Xue et al. (2012) identified known pathological variants such as *HBB* (c.20A>T; p.Glu7Val), which leads to increased resistance to malaria in heterozygotes but to sickle cell disease in homozygotes [confined to Africans (Yoruba, YRI) in whom there were 12 heterozygotes and 1 homozygote]. In addition, Xue et al. (2012) identified an *USH2A* variant (c.2138G>C; p.Gly713Arg), previously reported as being causal for Usher syndrome type 2, a recessive disorder characterized by combined deafness and blindness; three homozygotes were noted in the YRI. Manual curation of the HGMD-1000GP overlap revealed the presence of three types of DM: (1) plausible severe disease-causing variants, (2) variants convincingly causative for pathological conditions, yet quite compatible with adult life and (3) variants probably incorrectly assigned as disease causing. After applying various filtering criteria designed to enrich for true disease alleles, the list was reduced to 45 candidates (Xue et al. 2012). Of these putative disease alleles, 34 were present in the heterozygous state and were deemed likely to be present in asymptomatic carriers. Among the remaining 11 (Table 1), the 6 linked to dominant disorders were explicable in terms of either late onset (e.g. Gly507Arg in *MYBPC3*) or covert disease (Arg208His in *SERPIND1*). In similar vein, the presence of homozygotes for four of the five recessive disorders could be explicable in terms of late onset and/or reduced penetrance of disease. The *USH2A* mutation (Gly713Arg) was, however, intriguing: this variant was predicted to be damaging to the protein, and pathogenic in some populations but not in others (e.g. YRI). One explanation put forward to explain this apparent contradiction was that, in the YRI population, the *USH2A* locus is subject to copy number variation (Matsuzaki et al. 2009) that could provide functional complementation of the mutant gene. In the majority of cases, however, the most likely explanation for the absence of disease at the time of recruitment was considered to be the probable late onset of disease, although clinical penetrance was often variable, and some phenotypes, such as loose anagen hair syndrome [caused by Glu337Lys in *KRT75* (MIM 600628)], might not even be regarded as “diseases” sensu stricto. These factors notwithstanding, the findings of Xue et al. (2012) suggest that incidental findings which are potentially relevant to health and well-being might be made in as many as 11 % of individuals sequenced.

**Table 1:** Disease variants potentially capable of either causing dominant disease, or causing recessive disease and observed in the homozygous state, detected in 1000 Genomes Project participants (data from Xue et al. 2012)

Disease (MIM number)	Inheritance	Gene	HGVS cDNA mutation	Protein alteration	Total homozygotes	Total heterozygotes	Comments
Ataxia telangiectasia (MIM# 607585)	AR	*ATM*	NM_000051.3: c.4258C>T	p.Leu1420Phe	1	3	Low-penetrance breast cancer susceptibility allele
Usher syndrome type IIA (MIM# 276901)	AR	*USH2A*	NM_206933.2: c.2137G>C	p.Gly713Arg	3	22	Probable complex pathogenicity; neutral in YRI?
Nephronophthisis 4 (MIM# 606966)	AR	*NPHP4*	NM_015102.3: c.2542C>T	p.Arg848Trp	1	2	Growth retardation; adult-onset renal disease
Cushing syndrome (MIM# 607397)	AR	*MC2R*	NM_000529.2: c.833T>G	p.Phe278Cys	1	10	Hormonal disorder; variable gender-specific symptoms; variant functionally defective in vitro
Low-phospholipid-associated cholelithiasis (MIM# 171060)	AR	*ABCB4*	NM_000443.3: c.2363G>A	p.Arg788Gln	2	9	Adult onset
Cardiomyopathy, hypertrophic (MIM# 115197)	AD	*MYBPC3*	NM_000256.3: c.1519G>A	p.Gly507Arg	0	2	Late onset; incomplete penetrance
Glaucoma, primary open angle (MIM# 609887)	AD	*WDR36*	NM_139281.2: c.1586G>A	p.Arg529Gln	0	1	Adult onset; variant functionally defective in vitro
Colorectal cancer, nonpolyposis (MIM# 609310)	AD	*MLH1*	NM_000249.3: c.1742C>T	p.Pro581Leu	0	1	Adult onset; variant functionally defective in vitro
Renal cell carcinoma (MIM# 144700)	AD	*FLCN*	NM_144997.5: c.715C>T	p.Arg239Cys	0	1	Late onset
Heparin cofactor 2 deficiency (MIM# 612356)	AD	*SERPIND1*	NM_000185.3: c.623G>A	p.Arg208His	0	2	Deficiency state, but no overt disease; risk factor for thrombophilia
Lp(a) deficiency (MIM# 152200)	AD	*LPA*	NM_005577.2: c.4289+1G>A	essential splice site	0	5	Late onset; risk factor for heart disease

Reduced penetrance is one of several possible explanations for why some variants of putative pathological significance, listed in HGMD and/or Locus-specific Mutation Databases, nevertheless occur in apparently healthy individuals (Ashley et al. 2010; Bell et al. 2011; Xue et al. 2012; Golbus et al. 2012; Wang et al. 2013a; Kenna et al. 2013; Shen et al. 2013a). It is not hard to see why reduced penetrance might be much more common among described mutations than originally thought: whereas known pathological mutations have almost invariably been identified through retrospective analyses of families or well-defined groups of clinically symptomatic patients, relatively few prospective studies of asymptomatic carriers have so far been performed to derive estimates of penetrance (e.g. Jensen et al. 2013; Mavaddat et al. 2013). Indeed, establishing that a specific mutation identified in a particular patient with a given disease is the pathological lesion responsible for that individual’s clinical phenotype does not allow one automatically to judge whether this mutant genotype will invariably give rise to the same clinical phenotype in all other individuals harbouring it. This can only be established (or refuted) empirically by comprehensive, ideally prospective, studies of the genotype in question.

One fairly obvious reason why comparing the output of genome sequencing projects (e.g. the 1000 Genomes Project) with a comprehensive database of putatively pathological mutations (e.g. HGMD) is likely to generate a considerable number of potentially pathogenic mutations in the general population, is that many such mutations are quite frequent in the population at large. In particular, carrier frequencies for mutations underlying recessive conditions can often be quite high. Thus, the *ABCA4* Gly863Ala mutation causing Stargardt disease has a carrier frequency of 1.8 % in Europe (Maugeri et al. 2002), the *GJB2* 35delG mutation causing congenital deafness has a carrier frequency of 2.9 % in southern Europe (Gasparini et al. 2000), the *ERCC8* Tyr322Term mutation causing Cockayne syndrome has a carrier frequency of 6.8 % in Israeli Christian Arabs (Khayat et al. 2010) and the *SPG7* Ala510Val mutation associated with adult-onset neurogenetic disease has a carrier frequency of 3–4 % in the UK population (Roxburgh et al. 2013). Disease allele frequencies can be as high as 10 % in certain ethnic groups, e.g. Jews (Zlotogora et al. 2007; Ostrer and Skorecki 2013). A recent screen of the Korean population for 20 common mutations contributing to six autosomal recessive disorders yielded a combined carrier frequency of 6.7 % (Song et al. 2012). Screening an ethnically diverse US population sample (*N* = 364,890) for 87 different *CFTR* mutations responsible for causing cystic fibrosis yielded a combined carrier frequency of 2.6 % (Rohlfs et al. 2011). As part of the NHLBI-Go Exome Sequencing Project, a screen of 5,400 individuals from the general population for variants in eight long QT syndrome genes yielded a total of 33 different missense mutations (affecting 173 alleles), representing a carrier frequency of 3.2 % (Refsgaard et al. 2012). A similar screen for cardiomyopathy-associated gene variants yielded combined carrier frequencies for mutations reported to be disease associated of 25 % (1,474/5,810) for hypertrophic cardiomyopathy, 15 % (963/6,334) for dilated cardiomyopathy and (22 %) 1,393/6,359 for arrhythmogenic right ventricular cardiomyopathy (Andreasen et al. 2013); the high number of detected cardiomyopathy-associated gene variants suggests, however, that many are only modest disease modifiers or even non-pathogenic. Nishiguchi and Rivolta (2012) screened 46 complete genome sequences from the general population for mutations in 106 genes associated with recessively inherited retinal degeneration and identified null mutations in ten individuals (22 %). Finally, a routine screen of 23,453 individuals for 417 pathogenic mutations associated with a total of 108 recessive diseases concluded that 24 % of individuals were carriers of at least one disorder, whilst 5.2 % were carriers of two or more disorders (Lazarin et al. 2013). Clearly, there is a veritable abundance of actual and potential pathological variants segregating in the general population.

The examples that we cite in the text that follows and in the accompanying tables are by no means comprehensive and have been provided simply to illustrate the many and varied mechanisms that are already known to underlie the phenomenon of reduced penetrance in relation to clinical disorders. However, these examples also demonstrate that it is often very hard for authors, diagnosticians and mutation database curators alike to classify the pathogenicity of identified variants with any degree of certainty. Thus, is a given missense mutation a bona fide pathological lesion that exhibits greatly reduced penetrance, or is it essentially a neutral or near-neutral variant that occasionally finds itself in *cis* to other variants that are responsible for conferring the disease phenotype upon the individual concerned, or is it a variant with small effect that is sometimes present in affected individuals whose major causative lesions remain unknown?

## Incomplete penetrance in dominant and recessive conditions

A considerable number of autosomal dominant disorders are characterized by incomplete penetrance. Well-studied examples include the hair disease monilethrix (*KRT86*; De Cruz et al. 2012), congenital cataract (*GJA3*; Burdon et al. 2004), different types of retinitis pigmentosa (*PRPF8* and *PRPF31*; Maubaret et al. 2011; Saini et al. 2012), *LMNA* mutation-associated muscular phenotypes (Rankin et al. 2008) and long QT syndrome (Giudicessi and Ackerman 2013; Mathias et al. 2013).

One rather well-understood example of incomplete penetrance of a dominantly inherited mutation is factor V Leiden (*F5*, Arg534Gln; Arg506Gln in legacy nomenclature; rs6025) which occurs at polymorphic frequencies (2–5 %) in European populations, but is associated with a sixfold increased risk of venous thrombosis and a two- to threefold increased risk of pregnancy loss (Kujovich 2011). Despite these very evident disease associations, the vast majority of factor V Leiden carriers appear to be clinically unaffected. This could help to account for the high frequency of this variant in the general population, together perhaps with the survival advantage conferred by factor V Leiden carriership in various other clinical contexts including severe sepsis (Kerlin et al. 2003; van Mens et al. 2013). In similar vein, the asymptomatic (clinically covert) state is much more common than the clinically overt state in several other dominant disorders of haemostasis, including protein C deficiency (Tait et al. 1995; McColl et al. 1996), protein S deficiency (Dykes et al. 2001), antithrombin deficiency (Tait et al. 1994; McColl et al. 1996) and von Willebrand disease (Rodeghiero et al. 1987; Castaman et al. 2003). Thus, in disorders of haemostasis and thrombosis, even with well-characterized variants that are known to confer a significantly increased disease risk, the clinical penetrance is often so low that more healthy individuals carry the variant than those who actually manifest disease.

Reduced or incomplete penetrance has also been described for autosomal recessive disorders. Probably the best characterized example of incomplete penetrance in a recessive disorder is provided by the Cys282Tyr (rs1800562) mutation in the haemochromatosis (*HFE*) gene (Beutler 2003). The Tyr282 homozygous genotype is present in approximately 1 in 200 people of Northern European origin and is responsible for 80–90 % of hereditary haemochromatosis (Weiss 2010; Rochette et al. 2010). Although Tyr282 homozygosity displays a relatively high biochemical penetrance (i.e. iron accumulation), its clinical penetrance is low (McCune et al. 2006). Available data suggest that 38–50 % of Tyr282 homozygotes develop iron overload and 10–25 % develop some type of haemochromatosis-associated morbidity (Whitlock et al. 2006). However, these statistics conceal what appears to be a gender effect: large-scale studies of newly diagnosed Tyr282 homozygotes, in whom liver disease had been specifically assessed, revealed that disease manifested in 24–43 % of males, but only 1–14 % of females (Rossi et al. 2008). Various genetic modifiers have been identified as influencing the clinical expression of haemochromatosis. These include mutations in the *HAMP*, *HFE2* and *TFR2* genes and polymorphisms in the *BMP2*, *BMP4*, *CYBRD1*, *HP*, *LTA*, *MPO*, *TMPRSS6* and *TNF* genes (Milet et al. 2007; Rochette et al. 2010; Valenti et al. 2012; Pelucchi et al. 2012). In addition, several environmental modifiers (e.g. diet, alcohol intake) are also known to affect the penetrance of the *HFE* genotype.

In another recessive disorder, Gaucher disease, the most common *GBA* mutation, Asn370Ser (Asn409Ser in HGVS nomenclature; rs76763715), is also characterized by low penetrance and exhibits extensive phenotype heterogeneity even in the homozygous state (Sibille et al. 1993; Horowitz et al. 1998; Fairley et al. 2008). However, close examination of asymptomatic Ser370 homozygotes, serendipitously diagnosed by prenatal carrier screening, revealed a variety of previously unidentified disease manifestations indicating that the clinical penetrance of this disease genotype may be greater than previously appreciated (Balwani et al. 2010).

## Influence of mutation type on penetrance

Clinical penetrance is in part a function of the mutation(s) in question. For a given disease, some causal mutations may exhibit complete clinical penetrance, whereas other mutations in the same gene show incomplete or even very low penetrance. Thus, whereas the penetrance of the most common *CFTR* gene lesion, ΔPhe508 (rs113993960), in cystic fibrosis is very high, the penetrance of the *CFTR* Arg117His (rs78655421) mutation (in any allele combination) appears to be so low as to call into question its putative role as a pathological mutation (Thauvin-Robinet et al. 2009).

As yet, relatively few studies have been performed on low-penetrance mutations with a view to identifying the features responsible at the molecular level for their low penetrance. One exception is in retinoblastoma where it has been found that low-penetrance *RB1* mutations tend either to lead to a reduction in the amount of Rb protein produced (through promoter or splice site mutations) or yield a partially functional Rb molecule through missense mutation or in-frame deletion (Onadim et al. 1992; Kratzke et al. 1994; Bremner et al. 1997; Otterson et al. 1997; Scheffer et al. 2000; Genuardi et al. 2001; Harbour 2001; Klutz et al. 2002; Lefévre et al. 2002; Valverde et al. 2005; Sánchez-Sánchez et al. 2005; Sampieri et al. 2006; Gámez-Pozo et al. 2007; Park et al. 2008; Hung et al. 2011). Of particular interest is the category of temperature-sensitive mutations in the Rb pocket domain (ΔAsn480, Arg661Trp, Cys712Arg) whose ‘reversible fluctuations’ in a threshold level of Rb pocket-binding activity could be responsible for their characteristic low penetrance (Otterson et al. 1999). On the basis of studies performed to date, it would appear that a high proportion of *RB1* mutations with reduced penetrance are splice site mutations, although not all splice site mutations display low penetrance.

Some mutations are associated with specifically reduced penetrance as compared to other mutations of the same type in the same gene. For example, *BRCA1* Arg1699Gln is characterized by a cumulative risk of breast or ovarian cancer by the age of 70 years of only 24 % (Spurdle et al. 2012), much lower than for the average pathogenic *BRCA1* mutation (~71 %; van der Kolk et al. 2010). Individuals with *GJB2*-associated deafness who harbour two nonsense/truncating mutations exhibit a much more severe clinical phenotype, and hence are more likely to come to clinical attention, than those harbouring two missense mutations (Azaiez et al. 2004). This serves to illustrate that, in recessive disorders, the clinical penetrance of one mutation may be strongly influenced by the nature of the other mutation in *trans*. In Ehlers–Danlos syndrome type IV, however, null *COL3A1* mutations tend to exhibit lower penetrance than missense and splicing mutations (Leistritz et al. 2011); this is presumably because a faulty gene product can disrupt the entire triple helical collagen molecule, whereas a null mutation merely reduces the amount of normal collagen produced (Arnold and Fertala 2013). In the same vein, patients with heritable pulmonary arterial hypertension, due to missense mutations in the *BMPR2* gene, present earlier and with more severe disease than patients harbouring truncating mutations (Austin et al. 2009a). It is thought that the missense mutations are associated with stable *BMPR2* transcripts encoding BMPR2 protein which exerts a dominant negative effect on BMP signalling, thereby rendering missense mutations more detrimental than truncating mutations. The majority of *BMPR2* missense mutations were penetrant prior to the age of 36 years, whereas the majority of truncating mutations became penetrant only after the age of 36 years (Austin et al. 2009a). With mutations of the *TNFRSF1A* gene causing TNF receptor-associated periodic syndrome, missense mutations in cysteine residues have been reported to be more penetrant than missense mutations in non-cysteine residues (Aksentijevich et al. 2001; Aganna et al. 2001). Intriguingly, a *SOD1* Leu117Val missense mutation which yields a mutant protein indistinguishable from wild-type SOD1 (in terms of its activity, stability and folding) causes amyotrophic lateral sclerosis, but with unusually low penetrance and slow progression (Synofzik et al. 2012). Finally, clinical penetrance may vary not only with the mutation type, but also with the location of the mutation in the gene/protein (Jackson et al. 1999; Risch et al. 2001; Yatsenko et al. 2001; van der Werf et al. 2012; Ho et al. 2012).

In autosomal dominant hereditary pancreatitis, the penetrance of the *PRSS1* Arg122His mutation has been calculated to be 86 %, whereas that of *PRSS1* Ala16Val is of the order of 55–65 % (Grocock et al. 2010; Joergensen et al. 2010). It is thought that the comparatively low penetrance of this latter mutation may be related to its particularly mild biochemical phenotype. The Ala16Val substitution alters the *N*-terminal residue of the trypsinogen activation peptide, thereby increasing the rate of *N*-terminal processing by chymotrypsin C by ~5.8-fold (Szabó and Sahin-Tóth 2012). Since the activation peptide is released during the activation process, the Ala16Val mutation is absent from active trypsin and hence cannot influence trypsin function.

Missense mutations in the *MEFV* gene responsible for familial Mediterranean fever can differ quite dramatically in terms of their clinical penetrance. For example, Met694Val is generally characterized by high penetrance, whereas both Glu148Gln and Val726Ala exhibit reduced penetrance (Shohat and Halpern 2011). In this disorder, the carrier frequency is higher than would be expected from the prevalence of the disease, suggesting that the penetrance of pathogenic *MEFV* mutations may often be incomplete in the compound heterozygous state (Gershoni-Baruch et al. 2002; Zaks et al. 2003; Caglayan et al. 2010; Camus et al. 2012; Soriano and Manna 2012).

Double missense mutations in *cis* are not infrequently encountered in patients with an inherited disease. One of the two mutations may represent a hypomorphic (i.e. less functional) allele, as for example with the *GLA* Asp313Tyr occurring in *cis* to the pathogenic Gly411Asp in patients with Fabry disease (Yasuda et al. 2003). Double missense mutations in *cis* may however be associated with a highly variable clinical phenotype (e.g. *MEFV*, Pro369Ser/Arg408Gln as a cause of familial Mediterranean fever; Ryan et al. 2010). A low-penetrance missense mutation may be associated with a particularly severe clinical phenotype when it occurs in *cis* with a second known pathogenic mutation, e.g. *MYH7* Val606Met and Ala728Val in hypertrophic cardiomyopathy (Blair et al. 2001). Similarly, two missense mutations in *cis,* each individually exerting a comparatively mild or no effect on the clinical phenotype, can act in concert leading to a more severe effect on the phenotype than either acting alone (e.g. *CFTR* Arg347His and Asp979Ala in cystic fibrosis; Clain et al. 2001 or *RET* Cys634Tyr and Tyr791Phe resulting in pheochromocytoma with high penetrance; Toledo et al. 2010). By contrast, Brugnoni et al. (2013) have intriguingly claimed that two different *CLCN1* mutations do not give rise to myotonia congenita when they occur in *cis* on the same allele, although both lesions cause the disease when inherited on their own.

In diseases that exhibit locus heterogeneity, clinical penetrance may vary between mutations in different genes. For example, in pancreatitis, penetrance may vary from virtually 100 % in the case of the most common mutations in the cationic trypsinogen gene (*PRSS1*) gene, via an intermediate level for *SPINK1* and *CFTR* mutations, to the much more subtle risk conferred by the disease modifiers, namely variants in the chymotrypsin C (*CTRC*), calcium-sensing receptor (*CASR*) and anionic trypsin (*PRSS2*) genes, which can only be identified through large cohort studies (Lerch et al. 2010). It should, however, be noted that in cases where mutations in the *SPINK1* and *CASR* genes (Felderbauer et al. 2003) or *SPINK1* and *CFTR* genes (Masson et al. 2007) are co-inherited, chronic pancreatitis can ensue. Other such examples of digenic inheritance are discussed below (see *digenic mutations and disease penetrance*).

Reduced penetrance alleles are also characteristic of many triplet repeat expansion disorders. For example, in Huntington disease, the possession of intragenic (*HTT*) CAG repeats of 36–39 copies (in 0.01 % of controls and ~5 % of consultands) is often associated with reduced penetrance manifesting as a later onset of clinical symptoms (McNeil et al. 1997; Quarrell et al. 2007; Sequeiros et al. 2010; Panegyres and Goh 2011; Huntington Study Group COHORT Investigators 2012). Contractions of the expanded CAG repeat length below a certain threshold can occasionally be responsible even for the non-occurrence of Huntington disease in a given at-risk individual (Nahhas et al. 2009). However, it should be appreciated that a substantial proportion of the variance in age of onset in Huntington disease is due either to variation in genes other than *HTT* or in the environment (Wexler et al. 2004). Other repeat expansion disorders characterized by reduced penetrance of alleles of intermediate size include fragile X-associated tremor/ataxia syndrome (Jacquemont et al. 2004; Sévin et al. 2009), spinocerebellar ataxia types 10 (Alonso et al. 2006; Rankin et al. 2008) and 17 (Oda et al. 2004; Nolte et al. 2010), inherited prion disease (Kaski et al. 2011) and amyotrophic lateral sclerosis (Boeve et al. 2012; Ogaki et al. 2012).

## Modulating influence of additional allelic variants in *cis* or in *trans*

Some allelic variants may influence the expression of their host gene so as to alter the penetrance of a potentially pathological mutation in the same gene. Such modulatory variants may reside within exons, introns or regulatory regions. For example, the common Arg413Gln *F7* polymorphism (Arg353Gln in legacy nomenclature; rs6046), which serves to reduce the level of secreted coagulation factor VII by ~25 % (Arbini et al. 1994; Hunault et al. 1997), is over-represented among individuals with clinically symptomatic factor VII deficiency (Millar et al. 2000). This is consistent with the view that Arg413Gln is a functional polymorphism and that the presence of Gln413 increases the likelihood that an individual, whose haemostatic potential is already compromised by a heterozygous *F7* mutation, will come to clinical attention due to a bleeding diathesis. The same principle applies to the functional Arg202Gln *MEFV* polymorphism (rs224222) where the Gln202 allele occurs in the homozygous state at a disproportionately higher frequency (15 %) in familial Mediterranean fever patients than in normal controls (2.7 %) (Yigit et al. 2012). In similar vein, the T allele of a functional C/T polymorphism (rs11024595) in the promoter region of the *SAA1* gene is significantly over-represented in familial Mediterranean fever patients as compared with normal controls (Migita et al. 2013). The *MLH1* Lys618Ala mutation (AAG>GCG; rs35502531), initially supposed to be a benign polymorphism, has been found to be significantly over-represented in sporadic cancers associated with Lynch syndrome; *MLH1* Ala618 appears to have a reduced ability to bind PMS2, one of the MLH1 protein’s mismatch repair partners (Medeiros et al. 2012). Finally, the functional *KCNE1* Asp85Asn polymorphism (rs1805128), which occurs in the general population with a frequency of 0.8 %, occurs at a frequency of 3.9 % in long QT syndrome patients (Nishio et al. 2009).

Various reported examples of the modulation of the impact of pathogenic missense mutations by allelic single nucleotide polymorphisms (SNPs) are given in Table 2. Thus, the missense polymorphism Asp216His (rs1801968) in the *TOR1A* (DYT1) gene serves to moderate the clinical impact, in both *cis* and in *trans*, of the *TOR1A* c.904-906 del GAG mutation, the major mutation underlying early-onset dystonia (Kock et al. 2006; Risch et al. 2007; Martino et al. 2013). Similarly, in long QT syndrome, the genotype of a missense polymorphism (Lys897Thr; rs1805123) in the *KCNH2* gene appears to distinguish symptomatic from asymptomatic individuals carrying a low-penetrance Ala1116Val pathogenic mutation (Crotti et al. 2005). Another example of the modulatory effect of a missense polymorphism on disease allele penetrance is provided by the His558Arg substitution (rs1805124) in the *SCN5A* gene in a case of Brugada syndrome type 1 caused by compound heterozygous mutations (Asp1690Asn and Gly1748Asp) in *SCN5A*. Both mutations reduced the peak Na^+^ current density due to limited trafficking of the SCN5A protein towards the membrane, but Gly1748Asp also profoundly affected channel gating. The His558Arg polymorphism was found to be capable of rescuing the defective trafficking of SCN5A Asn1690 towards the membrane when present in *cis* to the pathological lesion (Núñez et al. 2013). Intriguingly, cotransfection with Asn1690, either alone or together with the modulatory His558Arg polymorphism, completely restored the gating defect associated with the pathogenic Gly1748Asp mutation in *trans*, although it only slightly rescued its trafficking.

**Table 2:** Examples of pathogenic microlesions whose penetrance has been found to be modulated by allelic SNPs

Disease	Gene	Pathological mutation	Modifying SNP	Reference
Factor VII deficiency	*F7*	Various	NM_000131.3: c.1238G>A Arg413Gln (rs6046)	Millar et al. (2000)
Brugada syndrome	*SCN5A*	NM_198056.2: c.5243G>A Gly1748Asp	NM_198056.2: c.1673A>G His558Arg (rs1805124)	Núñez et al. (2013)
Familial sick sinus syndrome	*SCN5A*	Various	NM_198056.2: c.1673A>G His558Arg (rs1805124)	Gui et al. (2010)
Early-onset dystonia	*TOR1A*	NM_000113.2: c.907-909delGAG	NM_000113.2: c.646G>C Asp216His (rs1801968)	Kock et al. (2006) and Risch et al. (2007)
Familial Mediterranean fever	*MEFV*	Various	NM_000243.2: c.605G>A Arg202Gln (rs224222)	Yigit et al. (2012)
Lynch syndrome	*MLH1*	Various	NM_000249.3: c.1852_1853delAAinsGC Lys618Ala (rs35502531)	Medeiros et al. (2012)
Long QT syndrome	*KCNH2*	NM_000238.3: c.3347C>T Ala1116Val	NM_000238.3: c.2690A>C Lys897Thr (rs1805123)	Crotti et al. (2005)
Long QT syndrome	*KCNH2*	NM_000238.3: c.1468G>A Ala490Thr	NM_000238.3: c.2690A>C Lys897Thr (rs1805123)	Zhang et al. (2008)
Creutzfeld–Jakob disease/fatal familial insomnia	*PRNP*	NM_000311.3: c.532G>A Asp178Asn	NM_000311.3: c.385A>G Met129Val (rs1799990)	Goldfarb et al. (1992) and Apetri et al. (2005)
Cardiac conduction abnormalities/sudden death	*SCN5A*	NM_198056.2: c.4262G>A Trp1421Term	NM_198056.2: c.3578G>A Arg1193Gln (rs41261344)	Niu et al. (2006)
Sudden unexplained death	*SCN5A*	NM_198056.2: c.2039G>A Arg680His	NM_198056.2: c.3308C>A Ser1103Tyr (rs7626962)	Cheng et al. (2011)
Syncope	*SCN5A*	NM_198056.2: c.5851G>T Val1951Leu	NM_198056.2: c.1673A>G His558Arg (rs1805124)	Shinlapawittayatorn et al. (2011)
Medullary thyroid carcinoma	*RET*	NM_020975.4: c.1996A>G Lys666Glu	NM_020975.4: c.2071G>A Gly691Ser (rs1799939)	Borrello et al. (2011)
Medullary thyroid carcinoma	*RET*	NM_020975.4: c.1597G>T Gly533Cys	NM_020975.4: c.74-126G>T (rs2565206)	Tamanaha et al. (2009)
Homocystinuria	*MTRR*	NM_002454.2: c.166G>A Val56Met	NM_002454.2: c.66G>A Ile22Met (rs1801394)	Gherasim et al. (2007)
Childhood absence epilepsy	*CACNA1H*	NM_021098.2: c.2318G>A Gly773Asp	NM_021098.2: c.2362C>T Arg788Cys (rs3751664)	Vitko et al. (2005)
Primary hyperoxaluria type 1	*AGXT*	NM_000030.2: c.731T>C Ile244Thr	NM_000030.2: c.32C>T Pro11Leu (rs34116584)	Santana et al. (2003)
Hereditary spherocytosis	*SPTA1*	NM_003126.2: c.7134G>A Gln2377Gln, alters splicing of exon 51	c.4339-99C>T (rs200830867)	Delaunay et al. (2004)
Erythropoietic protoporphyria	*FECH*	Various	NM_000140.3: c.315-48T>C (rs2272783)	Gouya et al. (2006)
Autosomal dominant osteopetrosis type II	*CLCN7*	Various	NM_001287.4: c.1252G>A Val418Met (rs12926089)	Chu et al. (2005)
Haemolytic uraemic syndrome	*CFH*	Various	NM_000186.3: c.2808G>T Glu936Asp (rs1065489)	Caprioli et al. (2003)
Hereditary spastic paraplegia	*SPAST*	NM_014946.3: c.1687G>A, (alters splicing of exon 15)	NM_014946.3: c.131C>T Ser44Leu (rs121908515)	Pantakani et al. (2008)
GM1 gangliosidosis	*GLB1*	NM_000404.2: c.601C>T Arg201Cys	NM_000404.2: c.1306C>T Leu436Phe (rs34421970)	Caciotti et al. (2003)
Primary cortisol resistance	*NR3C1*	NM_001018077.1: c.2035G>A Gly679Ser	NM_001018077.1: c.68G>A Arg23Lys (rs6190)	Raef et al. (2008)
Atopic dermatitis	*SPINK5*	NM_006846.3: c.2468dupA	NM_006846.3: c.1258G>A Glu420Lys (rs2303067)	Di et al. (2009)
Hyperinsulinism	*HADH*	NM_005327.4: c.636+471G>T	NM_005327.4: c.636+385A>G (rs732941)	Flanagan et al. (2013)

In passing, it is perhaps pertinent to note that interplay between functional SNPs may, depending upon the precise combination of alleles involved, also be sufficient to bring about disease in the absence of a pathogenic mutation sensu stricto. Thus, a particular allele of one SNP may contribute to pathogenesis, but only in the presence of specific allele of another SNP. These SNPs may be neighbouring, as in the case of Glu918Asp (rs16022) and Glu993Val (rs16023) in the *CACNA1A* gene, which appear to contribute to migraine susceptibility (D’Onofrio et al. 2009). In the same way, when they occur together in *cis*, two otherwise neutral missense polymorphisms in the *FMO3* gene [Glu158Lys (rs2266782) and Glu308Gly (rs2266780)] result in a decrease in FMO3 enzymatic activity that is sufficient to give rise to a mild form of trimethylaminuria (Akerman et al. 1999; Zschocke et al. 1999; D’Angelo et al. 2013). However, the interacting SNPs can also be located within different genes as in the case of Pro589Ser (rs1049296) in the transferrin (*TF*) gene and Cys282Tyr (rs1800562) in the haemochromatosis (*HFE*) gene which interact so as to increase the risk of Alzheimer disease (Robson et al. 2004; Kauwe et al. 2010). A further example of the combined effect of two unlinked SNPs is provided by an intronic SNP in the thrombospondin 2 (*THBS2*) gene (c.1478-8C>T; rs 9406328) and a missense SNP in the metalloproteinase 9 (*MMP9*) gene (Gln279Arg; rs17576), which together increase the risk of lumbar disc herniation (Hirose et al. 2008). This type of situation may occupy the middle ground between monogenic and complex disorders.

The modifying SNP may also be regulatory in nature and can serve to render the pathogenic coding mutation more or less deleterious (and hence more or less penetrant) depending upon whether the allele harbouring it is more or less expressed than the wild-type allele. An example of this is provided by the −30C>T variant (rs17249141) in the *LDLR* gene promoter that has been shown to act in concert with a low-penetrant missense mutation in *cis* so as to give rise to an unusually severe form of familial hypercholesterolaemia (Snozek et al. 2009). The modulating influence of regulatory SNPs on the penetrance of coding mutations located in *cis* appears to be a widespread phenomenon in medical genetics, with the high expressing SNP allele usually increasing the clinical penetrance of the linked coding mutation (Lappalainen et al. 2011). One regulatory SNP is thought to act as a low-penetrance cancer susceptibility factor in its own right: homozygosity for the intronic 309T>G *MDM2* variant (rs2279744), which leads to enhanced binding of the Sp1 transcription factor and *MDM2* up-regulation, appears to increase the risk for many types of tumour, presumably in concert with other lesions (Hu et al. 2007).

Introns may also harbour SNPs that are capable of modulating the clinical penetrance of a given pathogenic mutation. Thus, a short tract of five thymidines (5T) in intron 8 of the *CFTR* gene, found in ~10 % of individuals from the general population, can give rise to either congenital absence of the vas deferens (CAVD), non-classical cystic fibrosis or a normal phenotype when found in *trans* to a severe *CFTR* mutation (Kiesewetter et al. 1993; Cuppens et al. 1998). The number of TG repeats immediately adjacent to 5T is not only significantly associated with the level of alternative splicing of exon 9 of the *CFTR* gene (Cuppens et al. 1998; Niksic et al. 1999), but also influences clinical penetrance both in the context of cystic fibrosis and CAVD (Groman et al. 2004; Buratti et al. 2004; Lebo and Grody 2007). Another intronic modifying polymorphism is found in the *FECH* gene responsible for erythropoietic protoporphyria, an autosomal dominant disorder characterized by incomplete penetrance. This polymorphism (c.315-48T>C; rs2272783) modulates the use of a cryptic acceptor splice site, yielding an aberrantly spliced *FECH* mRNA which is degraded via nonsense-mediated mRNA decay (Gouya et al. 1999, 2002). The hypomorphic C allele increases the penetrance of erythropoietic protoporphyria when it occurs in *trans* to a pathogenic *FECH* mutation (Gouya et al. 2002, 2006). Finally, in a family with hyperinsulinism, a c.636+385A>G SNP (rs732941) in intron 5 of the *HADH* gene, which creates a cryptic acceptor splice site, acts in concert with a pathogenic *HADH* mutation (c.636+471G>T) in the same intron, which creates a cryptic donor splice site, to generate a 141-bp pseudoexon that leads to premature termination of translation (Flanagan et al. 2013).

A common C>T variant within an enhancer in intron 1 of the *RET* gene (rs2435357) serves to increase the clinical penetrance of *RET* coding sequence mutations (Emison et al. 2005). The T allele disrupts a SOX10-binding site, thereby reducing RET transactivation (Emison et al. 2010). A comparable example is provided by an intronic enhancer SNP (rs2596623) in the thyroid hormone receptor β (*THRB*) gene, which was found to be responsible for the pituitary cell-specific over-expression of a mutant thyroid hormone receptor β2 (Arg338Trp) in a case of pituitary cell-specific resistance to thyroid hormone (Alberobello et al. 2011).

Variants in the 3′ untranslated region (3′UTR) of the *KCNQ1* gene reportedly modify disease severity in individuals with type 1 long QT syndrome resulting from *KCNQ1* gene mutations (Amin et al. 2012). These variants serve to reduce *KCNQ1* gene expression, such that patients with one or more variants on their mutated *KCNQ1* alleles have a shorter QT interval and a milder clinical phenotype, whereas patients with the variants on their normal *KCNQ1* alleles exhibit significantly longer QT intervals and a more severe clinical phenotype. Another example of a modifying 3′UTR variant is provided by the G>A polymorphism (rs1799963) at position 20210 in the prothrombin (*F2*) gene, which increases the risk of venous thrombosis by enhancing *F2* mRNA 3′ end formation efficiency, thereby boosting thrombin formation (Gehring et al. 2001). This *F2* 20210G>A polymorphism has been claimed to interact with a common *F13A1* Val34Leu (rs5985) variant to confer a greatly increased risk of myocardial infarction (Butt et al. 2003). Some 3′UTR variants are located within microRNA-binding sites and may constitute low-penetrance risk factors for disease in their own right (Ahluwalia et al. 2009; Kontorovich et al. 2010; Qiu et al. 2011; Arnold et al. 2012). Finally, various SNPs in other non-coding RNAs (e.g. lincRNAs; Jendrzejewski et al. 2012; Kumar et al. 2013) appear to be disease associated and may therefore also influence disease penetrance.

## Influence of the gene expression level on mutation penetrance

Humans are characterized by marked inter-individual differences in the expression levels of their genes (Stranger et al. 2007; Skelly et al. 2009; Cowley et al. 2009; Cheng et al. 2012). Since gene expression is controlled by a combination of *cis*- and *trans*-acting regulatory factors, one means by which heritable differences in gene expression may be mediated is through polymorphism either of *trans*-acting regulatory (transcription) factors or of the *cis*-acting target sequences to which they bind. In the case of disease genes, such inter-individual variation in gene expression levels and patterns can influence the penetrance of pathological mutations. However, it should be appreciated that there are also substantial environmental and stochastic (non-genetic) components to gene expression that are likely to contribute to variable penetrance, even between monozygotic twins (Grundberg et al. 2012).

Differential allelic expression is a widespread phenomenon and is thought to be relevant to as many as 50 % of all human genes (Williams et al. 2007; Cheung and Spielman 2009; Palacios et al. 2009). In autosomal dominant conditions where the two alleles of the disease gene are expressed at different levels, this discrepancy can favour either the mutant or the wild-type allele and hence may influence clinical penetrance in either direction (de la Chapelle 2009). Thus, in pulmonary arterial hypertension, a disease caused by mutations in the bone morphogenetic protein receptor type 2 (*BMPR2*) gene, the penetrance of the *BMPR2* disease allele is dependent upon the level of expression of the wild-type *BMPR2* allele (Hamid et al. 2009a). Similarly, in erythropoietic protoporphyria, an autosomal dominant condition caused by mutations in the ferrochelatase (*FECH*) gene, the penetrance of the pathogenic *FECH* allele is influenced by the level of expression of the wild-type *FECH* allele (Gouya et al. 1999; 2002; Di Pierro et al. 2007). Other examples of autosomal dominant conditions where the degree of clinical penetrance is modulated by differential expression of the wild-type and mutant alleles include hereditary elliptocytosis (*SPTA1*, Wilmotte et al. 1993), Marfan syndrome (*FBN1*, Hutchinson et al. 2003), retinoblastoma (*RB1*, Taylor et al. 2007), colorectal cancer (*APC*, Yan et al. 2002; *TGFBR1*, Valle et al. 2008) and breast and ovarian cancer (*BRCA1*, Ginolhac et al. 2003).

Perhaps, the best understood example of penetrance depending upon the level of expression of the wild-type allele is retinitis pigmentosa type 11 (Utz et al. 2013). This autosomal dominant condition is caused by mutations in the pre-mRNA processing factor 31 (*PRPF31*) gene located on chromosome 19q13.42. The clinical penetrance of the underlying mutations has been shown to depend upon the level of wild-type *PRPF31* mRNA expression displayed by the patient (Vithana et al. 2003; Rivolta et al. 2006; Liu et al. 2008). Cells from asymptomatic carriers of *PRPF31* mutations express a higher level of the wild-type allele than cells from affected patients: high enough for the wild-type *PRPF31* mRNA level to lie within the range of the unaffected general population (Rivolta et al. 2006; Liu et al. 2008). The penetrance of *PRPF31* mutations is reduced by transcriptional repression mediated by the product of the CCR4-NOT transcription complex, subunit 3 (*CNOT3*) gene which is linked to *PRPF31* (McGee et al. 1997; Venturini et al. 2012). *PRPF31* expression has also been found to be strongly influenced by an unlinked eQTL on chromosome 14q21-q23 (Rio Frio et al. 2008). The penetrance of *PRPF31* mutations is therefore determined at least in part by a *trans*-acting modifier located on a different chromosome. The *trans*-acting alleles are inherited from the parent lacking the *PRPF31* mutation; these alleles are presumably present in the general population, but appear only to be relevant to disease when they modulate the penetrance of *PRPF31* mutations.

A slightly different scenario is exemplified by Schimke immune-osseus dysplasia (SIOD), a recessive condition, which appears to result from biallelic mutations in the *SMARCAL1* gene. Several examples of SIOD families with incomplete penetrance have been reported (Bökenkamp et al. 2005; Dekel et al. 2008; Elizondo et al. 2009). It has recently been shown that SMARCAL1, a protein involved in chromatin remodelling, influences the transcription level of many genes (Baradaran-Heravi et al. 2012). Although SMARCAL1 deficiency is insufficient in itself to cause SIOD in *Drosophila* and mouse models, the addition of environmental (viz. heat shock) and genetic insults affecting transcription can successfully recapitulate the pathophysiology of SIOD (Baradaran-Heravi et al. 2012). The penetrance of SIOD therefore appears to be dependent upon the magnitude of the alteration of gene expression consequent to SMARCAL1 deficiency.

In the case of a splicing mutation (c.291+1 G>A; rs71640277) in intron 3 of the *GH1* gene causing growth hormone deficiency type II, the expression levels of the mutant and wild-type alleles were found to correlate with the penetrance and expressivity of the deficiency state in different members of the same family (Hamid et al. 2009b). Some splicing mutations associated with low penetrance affect splicing in such a way that both normal-length and truncated transcripts are expressed from the same mutant allele, but presumably to different extents in different individuals (e.g. *RB1*, c.2211G>A; Schubert et al. 1997). Other such examples of reduced penetrance due to ‘leaky splicing’ involve splicing mutations in the *SPAST* gene causing hereditary spastic paraplegia (Svenson et al. 2001) and in the *BTK* gene causing X-linked agammaglobulinaemia (Kaneko et al. 2005). The reduced penetrance characteristic of some splicing mutations may also result from alternative splicing (Rave-Harel et al. 1997; Chiba-Falek et al. 1998; Nissim-Rafinia and Kerem 2005; Zinman et al. 2009; Szymanski et al. 2011; Cogan et al. 2012; Lee et al. 2012b) or internal translational start site initiation (Sánchez-Sánchez et al. 2007).

Special cases of differential allelic expression are of course provided by X-inactivation (Dobyns et al. 2004) and imprinting (Lo et al. 2003), both of which are discussed below.

## Allele dosage and its influence on penetrance

Formally, use of the term ‘autosomal dominant’ implies that the homozygotes exhibit the same or a similar clinical phenotype to the heterozygotes, as is the case in Huntington disease where the length of the expanded *HTT* CAG triplet repeat appears to be predictive of the age of onset irrespective of the presence or absence of a second expanded *HTT* allele (Lee et al. 2012a). However, in practice, for most ‘dominant’ human disorders in which homozygotes have been reported, their clinical symptoms tend to be significantly more severe than in the heterozygotes (Vogel and Motulsky 1997). This would seem to be especially true in the context of low-penetrance mutations such as those identified in the *SCN4A* and *CLCN1* genes, causing muscle channelopathies, conditions which are usually held to be transmitted in an autosomal dominant fashion. Patients homozygous for sodium channel mutations causing paramyotonia congenita (*SCN4A*, Ile1393Thr), hypokalemic periodic paralysis (*SCN4A*, Arg1132Gln) and myotonia congenita (*CLCN1*, Gly190Ser, Ile556Asn, Ala313Thr, Ile556Asn) display much more severe clinical features than patients heterozygous for these mutations (Plassart-Schiess et al. 1998; Arzel-Hézode et al. 2010; Shalata et al. 2010). The aforementioned mutations were also found to exhibit reduced penetrance in heterozygotes.

Mutations in the *RET* gene, associated with isolated Hirschsprung disease, are dominant loss-of-function mutations with incomplete penetrance and variable expressivity. Basel-Vanagaite et al. (2007) reported a c.1263+5G>A splicing mutation in the homozygous state in three females with severe Hirschsprung disease and in the heterozygous state in a male patient with short-segment Hirschsprung disease. In addition, a hypomorphic *RET*-predisposing allele, rs2435357, located in the first intron of the *RET* gene, was found in the heterozygous state in the male patient but not in the three affected females. Whilst the heterozygous c.1263+5G>A mutation is known to be low penetrance for short-segment Hirschsprung disease, the homozygous state is fully penetrant for total aganglionosis or long-segment Hirschsprung disease. Thus, the penetrance of *RET* gene mutations in Hirschsprung disease depends not only on the nature of the mutation but also on the allele dosage.

## Influence of copy number variation on mutation penetrance

Estimates of the clinical penetrance of recurrent pathogenic copy number variants (CNVs) vary quite widely, depending upon CNV size, genomic location and the disorder in question (Ben-Shachar et al. 2009; Vassos et al. 2010; Breckpot et al. 2011; Čiuladaitè et al. 2011; Hosak et al. 2012; Klopocki et al. 2012; Rosenfeld et al. 2013; Vaags et al. 2012; Weischenfeldt et al. 2013; Dabell et al. 2013; Carvill and Mefford 2013; Tropeano et al. 2013). In their study of children known to carry a CNV associated with intellectual disability and congenital abnormalities, Girirajan et al. (2012) reported synergy between multiple large CNVs leading to a particularly severe clinical presentation. Such a two-hit model, or ‘oligogenic heterozygosity’ as it has been termed, also appears to be characteristic of autism (Pinto et al. 2010; Schaaf et al. 2011b; Klei et al. 2012; Gau et al. 2012).

The penetrance of a given CNV may also be influenced by genetic variants in the vicinity. Thus, a submicroscopic deletion of 1q21.1 (encompassing the *RBM8A* gene) has been reported to interact with a low-frequency functional SNP in the regulatory region of the wild-type *RBM8A* allele to cause thrombocytopaenia with absent radii (Albers et al. 2012).

Several papers have now suggested that CNVs can also act as genetic modifiers of phenotype severity in a variety of different disease contexts (Beckmann et al. 2007; Chaudru et al. 2009; El-Hattab et al. 2010; Mulley et al. 2011; Jiang et al. 2011; Carvalho et al. 2012; Shen et al. 2013b). CNVs may influence the penetrance of a clinical phenotype indirectly as well as directly. For example, a mutant gene might be ‘covered’ by a CNV in a given individual, so that the expected clinical phenotype would be masked by the presence of an additional wild-type copy in *cis* to the gene in question. Consistent with this postulate, Ng et al. (2008) reported that ~30 % of nonsense SNPs occur in genes residing within segmental duplications, a proportion some threefold larger than that noted for synonymous SNPs. Genes harbouring nonsense SNPs were also found to belong to larger gene families (Ng et al. 2008) suggesting that some functional redundancy could also exist between paralogous human genes. In support of this idea, Hsiao and Vitkup (2008) reported that those human genes which have a homologue with >90 % sequence similarity are ~3 times less likely to harbour disease-causing mutations than genes with less closely related homologues. Hsiao and Vitkup (2008) interpreted their findings in terms of ‘genetic robustness’ against null mutations, with the duplicated sequences providing ‘backup’ by potentiating the functional compensation/complementation of homologous genes in the event that the latter acquired deleterious mutations. The capacity to be functionally compensated appears to vary, in the order non-disease genes > monogenic disease genes > polygenic disease genes (Podder and Ghosh 2011). One example of how a CNV can ameliorate the clinical phenotype is spinal muscular atrophy where an increased copy number of the *SMN2* gene can greatly reduce the severity of the disease caused by the homozygous deletion of the *SMN1* gene, because the *SMN2* gene, which lacks a splicing enhancer, can nevertheless generate some functional product thereby compensating functionally in a copy number-dependent fashion for the loss of the *SMN1* gene (Vitali et al. 1999; Harada et al. 2002; Wirth et al. 2006). Another example of how the clinical and/or phenotypic impact of a mutant gene can be nullified by a CNV is provided by a foetus that possessed paternal (Gln318Term) and maternal (Arg356Term) nonsense mutations of the *CYP21A2* gene but lacked the normal clinical sequelae of congenital adrenal hyperplasia; this was found to be due to a duplication of the *CYP21A2* gene on the paternal allele (Kleinle et al. 2009; Lekarev et al. 2013).

## Influence of modifier genes on disease penetrance

“For a so-called single gene disorder, there is one gene that may be primarily responsible for the pathogenesis with one or more independently inherited modifier genes that influence the phenotype. On the other hand, for a complex trait, the primacy of any individual gene is not perceptible, and the interaction of two or more independently inherited pairs of alleles, most likely influenced by additional modifier genes, results in the disease. The consequence of this conceptual framework is that there is no such thing as a ‘single’ gene disorder. In other words, there is no obvious clear distinction between simple Mendelian and complex traits: genetic diseases represent a continuum with diminishing influence from a single primary gene influenced by modifier genes, to increasingly shared influence by multiple genes”.

Dipple and McCabe (2000)

It is sometimes claimed that sickle cell anaemia is the simplest of all Mendelian disorders in that it is caused by one specific mutation (Glu7Val) in the β-globin (*HBB*) gene. However, this single-mutation monogenic disorder is not as simple as it might at first appear; indeed, it is characterized by marked clinical heterogeneity and incomplete penetrance of subphenotypes which is due in part to allelic variation and in part to variants in unlinked modifier genes (Steinberg and Sebastiani 2012). If we extrapolate from the archetypal example of sickle cell disease to other Mendelian disorders, it is not unreasonable to expect the action of modifier genes to be the rule rather than the exception. Indeed, variants in unlinked modifier genes have been reported to influence penetrance in a variety of different inherited diseases including pancreatitis (Khalid et al. 2006), breast cancer (Wolf et al. 2010; Wang et al. 2010a; Walker et al. 2010; Antoniou and Chenevix-Trench 2010; Esteban Cardeñosa et al. 2012; Harlid et al. 2012), Gaucher disease (Taddei et al. 2009; Zhang et al. 2012), retinitis pigmentosa (Rio Frio et al. 2008; Venturini et al. 2012), haemochromatosis (Krayenbuehl et al. 2010), hypertrophic cardiomyopathy (Daw et al. 2007), frontotemporal lobar degeneration (Finch et al. 2011) and amyloid polyneuropathy (Soares et al. 2005) among others. In familial late-onset Alzheimer disease, modifying loci may either influence the risk (Sleegers et al. 2009; Cruchaga et al. 2012) or the age of onset of disease (Wijsman et al. 2005; Marchani et al. 2010). Importantly, a significant excess of rare coding *APP*, *PSEN1* and *PSEN2* variants was noted in probands from late-onset Alzheimer disease families even though these variants did not actually co-segregate with the disease; this suggests that the variants in question may nevertheless serve to modulate the risk of disease (Cruchaga et al. 2012). An excess of rare variants as compared to controls has also been noted in patients with various other disorders including hypertriglyceridaemia (Johansen et al. 2012; Talmud 2007), hypertrophic cardiomyopathy (Lopes et al. 2013) and autism spectrum disorder (Mondal et al. 2012).

A typical example of a modifier gene in action is provided by long QT syndrome. The clinical penetrance of *KCNQ1* (potassium voltage-gated channel, KQT-like subfamily, member 1) mutations in this disorder is influenced by two coding sequence polymorphisms [Ser49Gly (rs1801252) and Arg389Gly (rs1801253)] in the *ADRB1* gene. Individuals homozygous for the Arg389 allele tend to have shorter QT intervals, whereas individuals homozygous for Ser49 tend to have longer QT intervals than those with other genotypes (Paavonen et al. 2007). Interestingly, those individuals doubly homozygous for Arg389 and Ser49 were found to be indistinguishable from the remainder of the patient cohort, both in terms of their QT intervals and in terms of clinical penetrance.

The minor allele of a variant in the complement receptor 1 (*CR1*) gene, Ser1610Thr (rs4844609), which has a population frequency of 0.02, is associated with episodic memory decline and susceptibility to Alzheimer disease (Keenan et al. 2012). However, this effect appears largely dependent upon an interaction with APOE-ε4, itself an important risk factor for Alzheimer disease (Mayeux et al. 1993).

Hirschsprung disease is one of the most complex genetic disorders in terms of the number of modifier genes (Garcia-Barcelo et al. 2009; Tang et al. 2010) known to influence the penetrance of its causative mutations, which has been estimated to be of the order of 50–70 % (Bolk et al. 2000). The best characterized of these modifier genes is the neuregulin 1 gene (*NRG1*; Tang et al. 2011, 2012a); however, most probably still remain to be identified. Some Bardet–Biedl syndrome patients also present with Hirschsprung disease. It appears that *RET*, the major gene involved in the aetiology of Hirschsprung disease, acts as a modifier of the Hirschsprung disease phenotype in Bardet–Biedl syndrome (de Pontual et al. 2007). Some families with Hirschsprung disease and Bardet–Biedl syndrome harbour mutations in their *BBS4*, *BBS5*, *BBS7* and *RET* genes (de Pontual et al. 2009). Sánchez-Mejías et al. (2009) reported a Hirschsprung disease family in which mutations in three different genes (*RET*, *NTRK3* and *EDN3*) contributed to the disease phenotype; the *RET* and *NTRK3* mutations were both necessary and sufficient to give rise to the clinical phenotype, whereas the *EDN3* mutation appeared to act as a modifier. More recently, copy number variations in various neurodevelopmental genes (*MAPK10*, *ZFHX1B*, *SOX2* and *NRG2*) have been shown to modify the penetrance of Hirschsprung disease (Jiang et al. 2011; Tang et al. 2012b). Taken together, these findings are consistent with an impact of both common and rare variants on the inheritance (and hence penetrance) of this highly complex disorder (Sánchez-Mejías et al. 2009; Núñez-Torres et al. 2011; Alves et al. 2013).

Table 3 lists a number of well-characterized examples of specific variants in modifier genes that serve to modulate the clinical penetrance of diseases caused by mutation(s) at unlinked loci.

**Table 3:** A selection of well-characterized examples of polymorphic variants in modifier genes that serve to modulate the clinical penetrance and/or severity of an inherited disease caused by mutation(s) at an unlinked locus

Disease	Primary disease gene	Modifier gene/variant	Reference
X-linked retinitis pigmentosa	*RPGR*	*IQCB1*/NM_001023570.2:c.1178T>A Ile393Asn (rs1141528)	Fahim et al. (2011)
X-linked retinitis pigmentosa	*RPGR*	*RPGRIP1L*/NM_015272.2:c.2231G>A Arg744Gln (rs2302677)	Fahim et al. (2011)
Retinal generation in ciliopathies	*RPGR* or *NPHP5*	*RPGRIP1L*/NM_015272.2:c.685G>A Ala229Thr (rs61747071)	Khanna et al. (2009)
Retinoblastoma	*RB1*	*MDM2*/NM_002392.4:c.14+309T>G (rs2279744)	Castéra et al. (2010)
Familial hypercholesterolaemia	*LDLR*	*PCSK9*/NM_174936.3:c.63_65dupGCT (rs35574083)	Abifadel et al. (2009)
Familial hypercholesterolaemia	*LDLR*	*APOB*/NM_000384.2:c.10580G>A Arg3527Gln (rs5742904)	Benlian et al. (1996) and Taylor et al. (2010)
Familial hypercholesterolaemia	*LDLR*	*CFH*/NM_000186.3:c.1204T>C Tyr402His (rs1061170)	Koeijvoets et al. (2009)
Familial hypercholesterolaemia	*LDLR*	*APOH*/NM_000042.2:c.1204T>C Leu266Val (rs4581)	Takada et al. (2003a)
Familial hypercholesterolaemia	*LDLR*	*GHR*/NM_000163.4:c.1630A>C Ile544Leu (rs6180)	Takada et al. (2003b)
Familial hypercholesterolaemia	*LDLR*	*EPHX2*/NM_001979.4:c.860G>A Arg287Gln (rs751141)	Sato et al. (2004)
Breast cancer	*BRCA2*	*RAD51*/NM_002875.4:c.-98G>C (rs1801320)	Antoniou et al. (2007)
Ovarian cancer	*BRCA1* or *BRCA2*	*IRS1*/NM_005544.2:c.2911G>A Gly971Arg (rs801278)	Ding et al. (2012)
Lynch syndrome	*MSH2* or *MLH1*	*RNASEL*/NM_021133.3:c.1385G>A Arg462Gln (rs486907)	Krüger et al. (2007)
Prostate cancer	*MSH2* or *MLH1*	*RNASEL*/NM_021133.3:c.1385G>A Arg462Gln (rs486907)	Krüger et al. (2005)
Lynch syndrome	*MSH2* or *MLH1*	*TP53*/NM_000546.3:c.215G>C Arg72Pro (rs1042522)	Krüger et al. (2007)
Cystic fibrosis	*CFTR*	*TGFB1*/NM_000660.4:c.29C>T Pro10Leu (rs1800470)	Drumm et al. (2005)
Familial pulmonary arterial hypertension	*BMPR2*	*TGFB1*/NM_000660.4:c.-1347C>T (rs1800469) & NM_000660.4:c.29C>T Leu10Pro (rs1800470)	Phillips et al. (2008)
Paget’s disease	*SQSTM1*	*TNFRSF11A*/NM_003839.2:c.575T>C Val192Ala (rs1805034)	Gianfrancesco et al. (2012)
X-linked variable immunodeficiency	*XIAP*	*CD40LG*/NM_000074.2:c.655G>A Gly219Arg (rs148594123)	Rigaud et al. (2011)
Haemochromatosis	*HFE*	*CYBRD1*/NM_024843.3:c.-399T>G (rs884409)	Constantine et al. (2009)
Parkinson’s disease	*GBA*	*MTX1*/NM_002455.3:c.187T>A Ser63Thr (rs760077)	Gan-Or et al. (2011)
Recessive dystrophic epidermolysis bullosa	*COL7A1*	*MMP1*/NM_002421.3:c.-1673delG (rs1799750)	Titeux et al. (2008)
Amyotrophic lateral sclerosis	*SOD1*	*CHGB*/NM_001819.2:c.1238C>T Pro413Leu (rs742710)	Gros-Louis et al. (2009)
Huntington disease	*HTT*	*HAP1*/NM_177977.2:c.1322C>T Thr441Met (rs4523977)	Metzger et al. (2008)
Fatal kernicterus	*G6PD*	*UGT1A1*/(TA)_6_/(TA)_7_ (rs8175347), HGVS nomenclature not available	Zangen et al. (2009)
Atypical haemolytic uraemic syndrome	*MCP* or *CFH*	*C4BPA*/NM_000715.3:c.719G>A Arg240His (rs45574833)	Blom et al. (2008)
Spinal muscular atrophy	*SMN1*	*SMN2*/NM_017411.3:c.859G>C Gly287Arg (rs121909192)	Prior et al. (2009)
Long QT syndrome	*KCNQ1*	*KCNH2*/NM_000238.3:c.2690A>C Lys897Thr (rs1805123)	Cordeiro et al. (2010)
Long QT syndrome	*KCNQ1*	*ADRB1*/NM_000684.2:c.145A>G Ser49Gly (rs1801252) & NM_000684.2:c.1165G>C Arg389Gly (rs1801253)	Paavonen et al. (2007)
Long QT syndrome	*KCNQ1*	*NOS1AP*/NC_000001.10:g.162029907A>T (rs4657139)	Crotti et al. (2009)
Familial venous thrombosis	*PROC*	*F5*/NM_000130.4:c.1601G>A Arg534Gln (rs6025; Factor V Leiden)	Koeleman et al. (1994); Gandrille et al. (1995) and Cafolla et al. (2012)
Familial venous thrombosis	*PROS1*	*F5*/NM_000130.4:c.1601G>A Arg534Gln (rs6025; Factor V Leiden)	Koeleman et al. (1995)
Familial venous thrombosis	*SERPINC1*	*F5*/NM_000130.4:c.1601G>A Arg534Gln (rs6025; Factor V Leiden)	Van Boven et al. (1996)
Hypertrophic cardiomyopathy	*MYBPC3* or *MYH7*	*CALM3*/NM_005184.2:c.−157T>A (rs150954567)	Friedrich et al. (2009)
Familial Mediterranean fever	*MEFV*	*SAA1*/NM_000331.4:c.−197C>T (rs11024595)	Migita et al. (2013)

The plastin 3 (*PLS3*) gene acts as a modifier of the clinical penetrance of autosomal recessive spinal muscular atrophy, caused by the homozygous deletion of the *SMN1* gene. Oprea et al. (2008) studied spinal muscular atrophy-discordant families with affected and unaffected *SMN1*-deleted siblings and found that all unaffected *SMN1*-deleted siblings were characterized by a high *PLS3* expression level in blood cells, considerably higher than in their affected counterparts (and despite high PLS expression being evident in only 5 % of healthy controls). Although it is still unclear whether *PLS3* expression is regulated by *cis*- or *trans*-acting factors, it would appear that high *PLS3* expression serves to rescue the spinal muscular atrophy patient from the detrimental effects of *SMN1* deletion by promoting axonogenesis through elevation of the level of F-actin (Oprea et al. 2008) and ultimately by improving neuromuscular transmission (Ackermann et al. 2013).

A unique kind of modifying effect is exemplified by α-thalassaemia/mental retardation syndrome, caused by mutations of the *ATRX* gene. The ATRX protein binds to variable number tandem repeat sequences (VNTRs) in the human genome, and genes associated with these VNTRs are dysregulated when *ATRX* is mutated (Law et al. 2010). Law et al. (2010) identified 917 ATRX targets in primary human erythroid cells, including one in the α-globin (*HBA*) locus. This ψδ VNTR was found to be highly polymorphic in terms of its length and acted as a length-dependent negative regulator of gene expression, its length serving to influence the degree of α-thalassaemia observed in an α-thalassaemia/mental retardation syndrome patient. Thus, the length of the ψδ VNTR could explain the incomplete penetrance of α-thalassaemia noted in individuals with identical *ATRX* mutations. A similar mechanism could underlie other genetic traits characterized by reduced penetrance.

## Digenic mutations and disease penetrance

In a typical autosomal Mendelian condition, a single mutation (dominant) or two mutations (recessive) at a specific locus give rise to a clinical phenotype. By contrast, digenic inheritance occurs in cases where the interaction of mutations in two different genes is required for the expression of the clinical phenotype. In this situation, a mutation in one copy of each gene is required for the full clinical phenotype to manifest. In the absence of one of the component mutations, the other mutation may be non-penetrant (digenic inheritance sensu stricto) or could be responsible for a less severe clinical phenotype (digenic inheritance sensu lato). At least 100 cases of probable and possible examples of digenic inheritance causing human inherited disease have been reported to date (Table 4).

**Table 4:** Examples of digenic mutations causing human inherited disease

Disease	Gene 1	Gene 2	Reference
Waardenburg syndrome type 2*	*MITF*	*PAX3* or *OCA3* or *TYR* or *GJB2*	Morell et al. (1997); Chiang et al. (2009); Yan et al. (2011) and Yang et al. (2013)
Retinitis pigmentosa*	*PRPH2*	*ROM1* or *RHO* or *PDE6B*	Kajiwara et al. (1994); Loewen et al. (2001); Sullivan et al. (2006) and Jin et al. (2008)
Retinitis pigmentosa	*RHO*	*PRPF31*	Lim et al. (2009)
Retinitis pigmentosa*	*PDE6B*	*GPR98*	Hmani-Aifa et al. (2009)
Progressive cone dystrophy	*CNGA3*	*CNGB3*	Thiadens et al. (2010)
Frontotemporal dementia*	*PSEN1*	*PRNP*	Bernardi et al. (2011)
Leber congenital amaurosis*	*RPE65*	*GUCY2D*	Silva et al. (2004)
Idiopathic hypogonadotropic hypogonadism*	*FGFR1*	*GNRHR* or *NELF*	Pitteloud et al. (2007)
Bilateral cystic renal dysplasia	*DACH1*	*BMP4*	Schild et al. (2013)
Glaucoma, early onset*	*MYOC*	*CYP1B1* or *LTBP2*	Vincent et al. (2002), Geyer et al. (2011) and Azmanov et al. (2011)
Severe insulin resistance	*PPARG*	*PPP1R3A*	Savage et al. (2002)
Usher syndrome type 2*	*PDZD7*	*GPR98*	Ebermann et al. (2010)
Usher syndrome type 1-associated deafness	*CDH23*	*PCDH15*	Zheng et al. (2005)
Hidrotic ectodermal dysplasia	*GJB2*	*GJA1*	Kellermayer et al. (2005)
Non-syndromic deafness*	*GJB2*	*GJB3*	Liu et al. (2009)
Hearing loss	*GJB2*	*SLC26A4*	Sagong et al. (2013)
Non-syndromic hearing loss associated with an enlarged vestibular aqueduct/Pendred syndrome	*KCNJ10*	*SLC26A4*	Yang et al. (2009)
Porphyria*	*CPOX*	*PPOX*	van Tuyll van Serooskerken et al. (2011)
Atypical haemolytic uremic syndrome	*CFI*	*CD46* or *C3* or *CFB* or *CFHR1*	Esparza-Gordillo et al. (2006), Westra et al. (2010) and Bresin et al. (2013)
Atypical haemolytic uraemic syndrome	*CFH*	*CD46* or *CFI* or *C3* or *THBD*	Sullivan et al. (2011), Bresin et al. (2013) and Fan et al. (2013)
Epidermolysis bullosa simplex*	*KRT14*	*KRT5*	Padalon-Brauch et al. (2012)
Junctional epidermolysis bullosa	*COL17A1*	*LAMB3*	Floeth and Bruckner-Tuderman (1999)
Long QT syndrome*	*KCNQ1*	*KCNH2* or *KCNE1* or *SCN5A*	Schwartz et al. (2003), Westenskow et al. (2004), Tester et al. (2005) and Itoh et al. (2010)
Long QT syndrome*	*KCNH2*	*SCN5A* or *KCNE1*	Schwartz et al. (2003), Westenskow et al. (2004) and Tester et al. (2005)
Long QT syndrome*	*SCN5A*	*SNTA1* or *KCNE1*	Westenskow et al. (2004) and Hu et al. (2013)
Haemochromatosis*	*HFE*	*HAMP* or *TFR2*	Merryweather-Clarke et al. (2003), Jacolot et al. (2004), Island et al. (2009), Altès et al. (2009) and Del-Castillo-Rueda et al. (2012)
Kallmann syndrome*	*PROK2*	*PROKR2*	Cole et al. (2008), Sarfati et al. (2010) and Shaw et al. (2011)
Kallmann syndrome	*NELF*	*KAL1* or *TACR3*	Xu et al. (2011) and Quaynor et al. (2011)
Kallmann syndrome	*PROKR2*	*KAL1*	Dodé et al. (2006), Canto et al. (2009) and Shaw et al. (2011)
Kallmann syndrome	*KAL1*	*TACR3* or *WDR11* or *CHD7*	Quaynor et al. (2011) and Shaw et al. (2011)
Normosmic idiopathic hypogonadotrophic hypogonadism	*GNRH*	*KAL1*	Quaynor et al. (2011)
Normosmic idiopathic hypogonadotrophic hypogonadism	*WDR11*	*GNRHR*	Quaynor et al. (2011)
Normosmic idiopathic hypogonadotrophic hypogonadism	*FGFR1*	*GNRHR* or *PROKR2* or *FGF8* or *KAL1* or *GPR54*	Raivio et al. (2009), Sykiotis et al. (2010) and Shaw et al. (2011)
Systemic amyloid A amyloidosis	*TNFRSF1A*	*MEFV*	Cigni et al. (2006) and Mereuta et al. (2013)
Familial hypercholesterolaemia*	*LDLR*	*PCSK9*	Pisciotta et al. (2006), Noguchi et al. (2010) and Bertolini et al. (2013)
Familial hypercholesterolaemia	*LDLR*	*APOB*	Bertolini et al. (2013)
Familial hypercholesterolaemia	*LDLR*	*LDLRAP1*	Tada et al. (2011)
Severe congenital neutropenia	*ELANE*	*G6PC3* or *HAX1*	Germeshausen et al. (2010)
McArdle’s disease*	*PYGM*	*CPT2*	Vockley et al. (2000)
Parkinson’s disease, early onset	*PINK1*	*PARK2* or *PARK7*	Tang et al. (2006) and Funayama et al. (2008)
Parkinson’s disease	*LRRK2*	*PRKN*	Dächsel et al. (2006)
Emery–Dreifuss muscular dystrophy*	*LMNA*	*DES*	Muntoni et al. (2006)
Joubert syndrome and nephronophthisis*	*NPHP1*	*NPHP6*	Tory et al. (2007)
Axenfeld–Rieger syndrome	*FOXC1*	*PITX2*	Kelberman et al. (2011)
Cortisone reductase deficiency	*HSD11B1*	*H6PD*	Draper et al. (2003) and San Millán et al. (2005)
Hypertrophic cardiomyopathy*	*MYBPC3*	*TNNT2* or *TNNI3* or *MYH7* or *TPM1*	Richard et al. (2003), Van Driest et al. (2004) Ingles et al. (2005), Millat et al. (2010), Kubo et al. (2011) and Zou et al. (2013)
Hypertrophic cardiomyopathy*	*MYH7*	*TNNT2* or *MYL2* or *TNNI3* or *ACTC1*	Millat et al. (2010) and Zou et al. (2013)
Restrictive cardiomyopathy*	*MYL2*	*MYL3*	Caleshu et al. (2011)
Rasopathy phenotype with severe hypertrophic cardiomyopathy	*PTPN11*	*SOS1*	Fahrner et al. (2012)
Arrhythmogenic right ventricular cardiomyopathy	*DES*	*PKP2*	Lorenzon et al. (2013)
Arrhythmogenic right ventricular cardiomyopathy*	*DES*	*DSG2*	Rasmussen et al. (2013)
Arrhythmogenic right ventricular cardiomyopathy	*PKP2*	*DSP* or *DSG2* or *PKP4* or *DSC2*	Xu et al. (2010)
Arrhythmogenic right ventricular dysplasia/cardiomyopathy	*DSG2*	*DSC* or *PKP2*	Bhuiyan et al. (2009) and Nakajima et al. (2012)
Familial dilated cardiomyopathy*	*LMNA*	*TTN*	Roncarati et al. (2013)
Dent’s disease	*CLCN5*	*OCRL*	Addis et al. (2013)
Amyotrophic lateral sclerosis	*SOD1*	*CNTF*	Giess et al. (2002)
Amyotrophic lateral sclerosis	*VAPB*	*C9orf72*	van Blitterswijk et al. (2012b)
Dravet syndrome	*PCDH19*	*TSPYL4*	Kwong et al. (2012)
Dravet syndrome*	*SCN9A*	*SCN1A*	Singh et al. (2009)
Dravet syndrome*	*CACNA1A*	*SCN1A*	Ohmori et al. (2013)
Severe myoclonic epilepsy	*CACNB4*	*SCN1A*	Ohmori et al. (2008)
Severe myoclonic epilepsy	*POLG*	*SCN1A*	Bolszak et al. (2009)
Progressive external ophthalmoplegia	*POLG*	*SLC25A4*	Galassi et al. (2008)
Bartter syndrome	*CLCNKA*	*CLCNKB*	Nozu et al. (2008)
Chronic pancreatitis	*SPINK1*	*CASR* or *CFTR* or *CTRC* or *PRSS1*	Felderbauer et al. (2003), Masson et al. (2007), Tzetis et al. (2007), Schneider et al. (2011), LaRusch et al. (2012) and Rosendahl et al. (2013)
Oculocutaneous albinsim	*OCA2*	*TYRP1* or *SLC45A2* or *TYR*	Chiang et al. (2008) and Wei et al. (2013)
Oculocutaneous albinsim	*TYR*	*SLC45A2*	Wei et al. (2013)
Cystinuria	*SLC3A1*	*SLC7A9*	Font-Llitjós et al. (2005)
Transposition of the great arteries	*ZIC3*	*FOXH1* or *NKX2*-*5*	De Luca et al. (2010)
Congenital heart disease	*MYH6*	*NKX2*-*5* or *GATA4*	Granados-Riveron et al. (2012)
Charcot–Marie–Tooth disease*	*PMP22*	*ABCD1* or *LITAF*	Meggouh et al. (2005), Hodapp et al. (2006)
Charcot–Marie–Tooth disease	*GJB1*	*EGR2*	Chung et al. (2005)
Charcot–Marie–Tooth disease*	*GDAP1*	*MFN2*	Vital et al. (2012)
Refractory auto-inflammatory syndrome	*TNFRSF1A*	*CIAS1*	Touitou et al. (2006)
Short-rib polydactyly syndrome type 2	*NEK1*	*DYNC2H1*	Thiel et al. (2011)
Maturity-onset diabetes of the young	*HNF1A*	*HNF1B*	Karges et al. (2007)
Maturity-onset diabetes of the young	*HNF1A*	*HNF4A*	Forlani et al. (2010) and Shankar et al. (2013)
Polycystic kidney disease*	*PKD1*	*PKD2*	Pei et al. (2001) and Dedoussis et al. (2008)
Hyperimmunoglobulinaemia D and periodic fever syndrome	*MVK*	*TNFRSF1A*	Hoffmann et al. (2005)
Obesity, hyperinsulinaemia and insulin resistance	*TCF1*	*NROB2*	Tonooka et al. (2002)
Progressive external ophthalmoplegia	*POLG*	*C10orf2*	Van Goethem et al. (2003)
Neuronal ceroid lipofuscinosis	*POLG*	*CLN5*	Staropoli et al. (2012)
Chronic lung disease	*SFTPC*	*ABCA3*	Bullard and Nogee (2007)
Lafora disease	*EPM2B*	*PPP1R3C*	Guerrero et al. (2011)
Congenital erythropoietic porphyria	*UROS*	*ALAS2*	To-Figueras et al. (2011)
Familial venous thrombosis*	*PROC*	*PROS1*	Formstone et al. (1996), Brenner et al. (1996), Boinot et al. (2003), Knoll et al. (2001) and Hayashida et al. (2003)
Familial venous thrombosis*	*PROC*	*SERPIND1*	Bernardi et al. (1996)
Breast cancer*	*BRCA1*	*BRCA2*	Leegte et al. (2005), Lavie et al. (2011) and Heidemann et al. (2012)
Breast cancer	*BRCA1*	*PALB2*	Pern et al. (2012)
Multiple tumours of different types	*BRCA1*	*MLH1*	Pedroni et al. (2013)
Familial pulmonary arterial hypertension	*BMPR2*	*THBS1*	Maloney et al. (2012)
Hereditary nonpolyposis colorectal cancer	*MUTYH*	*MSH6*	Van Puijenbroek et al. (2007) and Giráldez et al. (2009)
Colorectal cancer	*EPCAM*	*MSH2*	Li-Chang et al. (2013)
Colorectal cancer, juvenile onset*	*APC*	*MSH2*	Uhrhammer and Bignon (2008)
Autoimmune lymphoproliferative syndrome	*FAS*	*CASP10*	Cerutti et al. (2007)
Autoimmune lymphoproliferative syndrome*	*FAS*	*PRF1*	Clementi et al. (2004)
Steroid-resistant focal segmental glomerulosclerosis	*NPHS2*	*NPHS1* or *CD2AP*	Löwik et al. (2008)
Severe infantile liver disease	*AKR1D1*	*SKIV2L*	Morgan et al. (2013)
Ataxia, dementia and hypogonadotropism	*RNF216*	*OTUD4*	Margolin et al. (2013)
Paediatric inflammatory bowel disease	*NOD2*	*GSDMB* or *ZNF365* or *ERAP2* or *SEC16A* or *GMPBB*	Christodoulou et al. (2013)
Paediatric inflammatory bowel disease	*BACH2*	*IL10*	Christodoulou et al. (2013)
Hutchinson-Gilford progeria syndrome	*ZMPSTE24*	*LMNA*	Denecke et al. (2006)

In this table, we considered only those examples of digenic mutations that are unlikely to be merely coincidental and which are predicted to affect genes that are both functionally associated with the disease in question

* Patients, with heterozygous mutations affecting two different genes, exhibiting earlier disease onset or a more severe clinical phenotype than either of their singly heterozygous parents or their siblings

Digenic inheritance may occur as a result of mutations in genes encoding different subunits of the same multimeric protein (e.g. *PRPH2* and *ROM1*), an oligomeric protein complex (e.g. *KCNJ10* and *SLC26A4*; *KRT14* and *KRT5*) or simply two proteins that interact functionally with each other (*CDH23* and *PCDH15*; *DSG2* and *DSC2*; *PARK7* and *PINK1*). However, mutations in receptor/ligand pairs can also give rise to digenic inheritance (e.g. *PROK2* and *PROKR2*). Alternatively, digenic inheritance can involve mutations located in different genes, but compromising the same regulatory (e.g. *HFE* and *HAMP*), biosynthetic (e.g. *ZMPSTE24* and *LMNA*; *CPOX* and *PPOX*; *MYOC* and *CYP1B1*) or degradative (e.g. *PCSK9* and *LDLR*) pathway. Finally, the combination of a mutation in a transcription factor with a mutation in a target gene of that transcription factor can also serve to reduce the amount of the protein in question to a level sufficient to cause a disease phenotype (e.g. *MITF* and *TYR*; *FOXC1* and *PITX2*).

In practice, it is not always clear if a given situation constitutes true digenic inheritance (e.g. Kajiwara et al. 1994) or whether it is simply the coinheritance of two mutations in different genes (e.g. Gruber et al. 2009; Serrano-Fernández et al. 2009; Ekvall et al. 2011). In the former case, the expression of the disease phenotype actually requires the presence of both gene lesions. In the latter case, coinheritance of the two gene lesions may serve to aggravate the clinical phenotype, but each lesion is independently associated with its own characteristic clinical sequelae. In true digenic inheritance, mutations in both genes must be present for the genetic disorder to be manifest. Since many of the disorders reported (in Table 4) to be characterized by digenic inheritance also have monogenic forms in which just one of the two genes has been mutated (whether in the heterozygous or homozygous/compound heterozygous state), it is unclear how many of the examples listed really represent digenic inheritance sensu stricto. However, in many of the listed examples, the doubly heterozygous probands exhibit earlier onset or a more severe clinical phenotype than their singly heterozygous relatives (although this is not invariably so; Marras et al. 2010). Further, whereas the truly ‘digenic’ patients tend to be characterized by complete penetrance, the monogenic disease genotypes often exhibit reduced penetrance as in e.g. normosmic idiopathic hypogonadotrophic hypogonadism (see Table 4).

In some of the cases listed in Table 4, the digenic inheritance may be confined to a single proband and hence it is not always straightforward to distinguish true digenic inheritance from the chance coinheritance of two mutations in unlinked genes. This notwithstanding, the requirement for the involvement of a second mutated gene may depend upon the specific mutations that are segregating in the pedigrees. If digenic inheritance eventually turns out to be more frequent than previously appreciated, it could provide yet another reason why some potentially pathogenic alleles are present in the general population in the absence of overt disease. At the very least, in many cases the clinical penetrance of the condition in question is likely to be greater when two relevant genes have been functionally compromised by mutation than if only one had been mutated.

## Oligogenic inheritance and its implications for disease penetrance

Triallelic inheritance has been described as being “a bridge between Mendelian and multifactorial traits” (Eichers et al. 2004). There are a burgeoning number of reported examples of digenic triallelic inheritance including nephronophthisis (Hoefele et al. 2007), venous thrombosis (Formstone et al. 1996; Brenner et al. 1996) and cortisone reductase deficiency (Draper et al. 2003). It would not be altogether surprising, in conditions where digenic or even trigenic inheritance has been reported, if the individual component mutations were found to exhibit a reduced clinical penetrance as compared to mutations underlying the monogenic forms of the disease.

In some disorders, incomplete penetrance of a particular mutation can be due to the oligogenic nature of the disease and hence to the requirement for multiple genes to be mutated for the condition in question to manifest. An inherited predisposition to cancer can be monogenic, but is also very likely to have an oligogenic aetiology in many instances (Fearnhead et al. 2004; Koren-Michowitz et al. 2005; Okkels et al. 2006; Küry et al. 2008; Wasielewski et al. 2010; Martinez and Kolodner 2010; Plon et al. 2011; Morak et al. 2011; Gracia-Aznarez et al. 2013). In amyotrophic lateral sclerosis, van Blitterswijk et al. (2012a) detected *FUS* and *TARDBP* mutations in combination with *ANG* mutations, and *C9orf72* repeat expansions with *TARDBP*, *SOD1* and *FUS* mutations. At least five relatively common polymorphisms in four different genes, *CFB* (Arg32Gln, rs641153), *C2* (Glu318Asp, rs9332739), *CFH* [Tyr402His (rs1061170) and non-coding variant rs1410996] and *ARMS2* (Ala69Ser, rs10490924), interact so as to confer increased risk of age-related macular degeneration (Maller et al. 2006). Common polymorphisms in the *LEPR* (Gln223Arg, rs1137101) and *ADRB2* [Arg16Gly (rs1042713) and Gln27Glu (rs1042714)] genes jointly confer increased risk of obesity even though none of these polymorphisms exhibits a significant influence on their own (Pereira et al. 2011). Other examples of oligogenic inheritance, involving the mutation or polymorphism of multiple unlinked genes in the same individual, include isolated gonadotropin-releasing hormone deficiency (*KAL1*, *PROK2* and *NELF*; Sykiotis et al. 2010), hypertrophic cardiomyopathy (*MYH7*, *MYBPC3*, *TNNI3* and *TNNT2*; Girolami et al. 2010; Lopes et al. 2013), iminoglycinuria (*SLC36A2*, *SLC6A20*, *SLC6A18*, *SLC6A19*; Bröer et al. 2008), long QT syndrome (*KCNH2*, *SCN5A* and *KCNE1*; Yoshikane et al. 2013), chronic pancreatitis (*SPINK1*, *CFTR* and *CTRC*; Rosendahl et al. 2013), atypical haemolytic uraemic syndrome (*CFH*, *CD46* and *CFI*; Roumenina et al. 2012; Bresin et al. 2013), Parkinson disease (*LRRK2*, *SNCA*, *MAPT*, *GBA*, *BST1*, *PARK16*; Wang et al. 2012), acrocallosal syndrome (*KIF7*, *AHI1*, *BBS2* and *BBS4*; Walsh et al. 2013), autism spectrum disorders (Neale et al. 2012) and a low plasma level of HDL cholesterol, a major risk factor for atherosclerosis (Cohen et al. 2004; Wang et al. 2008; Johansen and Hegele 2012).

One of the best characterized oligogenic disorders is Bardet–Biedl syndrome (BBS) where at least 17 genes are known to contribute to the clinical phenotype, and the severity of the disease phenotype may vary as a result of the interaction of mutations in different BBS genes. Although mutations at more than one locus have often been found to segregate with the disease, thereby modulating both its penetrance and expressivity (Badano et al. 2006; Zaghloul and Katsanis 2009; Cardenas-Rodriguez et al. 2013), the jury is still out in relation to claims of digenic triallelic inheritance in BBS (Katsanis et al. 2001; Beales et al. 2003; Badano et al. 2003; Fauser et al. 2003; Smaoui et al. 2006; Chen et al. 2011; Abu-Safieh et al. 2012). This notwithstanding, some common BBS gene variants appear to be detrimental to protein function and may well interact with the much rarer pathogenic BBS mutations so as to influence the severity of the BBS phenotype (Zaghloul et al. 2010).

Some idea of the likely complexity of genotype–phenotype relationships in complex disease has come from the comparative exome sequencing of 237 ion channel genes performed in sporadic idiopathic epilepsy patients and unaffected controls (Klassen et al. 2011). Both rare and common variants were identified in the two groups. Although these variants were more numerous in the patient group, they were not found to be predictive of disease: as the authors opined, “absolute numerical counts of SNP burden hold little predictive value as a global pathogenic measure”. However, 51 % of cases and 14 % of controls had ≥2 non-synonymous variants in their sodium channel genes. In similar vein, 24 % of cases and 6 % of controls had ≥2 non-synonymous variants in their GABA receptor alpha genes (Klassen et al. 2011). Such findings are quite consistent with an oligogenic model of sporadic epilepsy (Dibbens et al. 2007). What is required here, however, is not a simple variant number count, but rather an assessment, on an individual basis, of the net effect of an oligogenic variant profile (requiring ascertainment of the various gains or losses of function associated with specific variants and computed with an eye to the nature of potential joint effects) on a clinically or phenotypically relevant output measure such as the electrical signature of a cell type or brain region.

Perhaps, the best characterized oligogenic disorder to date is familial venous thrombosis. The risk of venous thromboembolism is known to be increased in patients who carry more than one genetic variant disrupting the 100+ genes of the ‘hemostaseome’ (Fechtel et al. 2011). Thus, 19 % of symptomatic individuals harbouring a protein C (*PROC*) gene mutation were also found to be heterozygous for factor V Leiden (*F5* Arg534Gln; Koeleman et al. 1994), a functional polymorphism which occurs at a frequency of 2–5 % in European populations. In a replication study, 9.5 % of venous thrombosis patients were found to carry both mutations (Gandrille et al. 1995) suggesting that their co-occurrence increases the likelihood of their coming to clinical attention. Similar findings have been noted in families with protein S (*PROS1*) deficiency; among symptomatic individuals, 38 % also carried the factor V Leiden mutation (Koeleman et al. 1995). Likewise, coinheritance of antithrombin (*SERPINC1*) deficiency and factor V Leiden not only increases clinical penetrance, but also reduces the age of clinical presentation (van Boven et al. 1996). In a larger-scale study involving 132 thrombophilic families, the risk of thrombosis was increased and the age of onset lowered in cases of double heterozygosity for two gene variants (combinations of variants in *PROC*, *PROS1*, *F5* and *F2*) as compared to individuals carrying single variants of these genes (Tirado et al. 2001). ABO blood group is also known to modify the risk of venous thrombosis in individuals with hereditary thrombophilia through an influence on the plasma levels of factor VIII and the factor VIII carrier protein, von Willebrand factor (Tirado et al. 2005; Nossent et al. 2006; Cohen et al. 2012). Such studies provide strong circumstantial support for the joint impact of multiple mutations in thrombotic disease (Martinelli et al. 2008). The elevation of risk for each individual variant is, however, low, and incomplete penetrance is evident for all prothrombotic variants. This means that the vast majority of individuals bearing these variants do not suffer from thrombotic disease. It is nevertheless reasonable to suppose that patients with recurrent venous thrombosis will tend to have a greater number of prothrombotic variants than those who have experienced a single thrombotic event, with those individuals who never experienced thrombosis harbouring even fewer prothrombotic variants (Fechtel et al. 2011). Since the number of genes known to influence haemostasis is large and the number of variants with potential impact larger still, we may expect that a substantial number of different variant combinations will be capable of conferring an increased risk. This genetic risk will accompany each prothrombotic challenge (such as pregnancy, long haul air travel, contraceptive pill usage and immobilization after surgery), with the greatest risk of venous thrombosis accruing to those possessing the largest number of prothrombotic variants in their genomes (Fechtel et al. 2011). Our task is to come to understand how specific DNA sequence changes in the large number of genes known to play a role in haemostasis and thrombosis act either synergistically or antagonistically so as to confer disease predisposition upon the individual, thereby influencing the clinical penetrance, by shifting their haemostatic balance towards either a prothrombotic or anticoagulant phenotype (Franchini and Mannucci 2009; Westrick and Ginsburg 2009; Fechtel et al. 2011).

## Influence of sex on penetrance

The sex dependence of the penetrance of inherited mutations has been reported in a variety of different heritable disorders including haemochromatosis (*HFE*; Rossi et al. 2008), hypertrophic cardiomyopathy (*MYBPC3*, *MYH7*; Michels et al. 2009; Page et al. 2012), arrhythmogenic right ventricular dysplasia/cardiomyopathy (*PKP2*; Dalal et al. 2006), long QT syndrome (*KCNQ1*, *KCNH2*, *SCN5A*; Zareba et al. 2003), hypokalaemic periodic paralysis (*CACNA1S*; Kawamura et al. 2004; Li et al. 2012; *SCN4A*; Ke et al. 2013), familial pulmonary arterial hypertension (*BMPR2*; Austin et al. 2009b), hereditary spastic paraplegia (*SPAST*; Mitne-Neto et al. 2007), hereditary dystonia/dopa-responsive dystonia (*GCH1*; Furukawa et al. 1998), cardiac disease (*LMNA*; van Rijsingen et al. 2013), Hirschsprung disease (*RET*; Emison et al. 2005), autism spectrum disorder (*SHANK1*; Sato et al. 2012), amyotrophic lateral sclerosis (*C9ORF72*; Le Ber et al. 2013; Williams et al. 2013) and familial obesity (*SHP*; Yang et al. 2010). A male-biased effect on the penetrance of duplications and deletions at 16p13.11 is evident in a range of neorodevelopmental conditions including autism, attention deficit hyperactivity disorder, intellectual disability and schizophrenia (Tropeano et al. 2013). In the case of familial pulmonary arterial hypertension, both genetic and metabolic marker data were consistent with a modifying role for variation in oestrogens and/or oestrogen metabolism upon disease risk (Austin et al. 2009b). The low penetrance of hypokalaemic periodic paralysis due to *SCN4A* mutations in females is also likely to be due to the effect of oestrogens (Ke et al. 2013).

Allelic variation may also influence the clinical phenotype in a sex-specific fashion. Thus, Lahtinen et al. (2011) reported that the common *KCNE1* Asp85Asn (rs1805128) polymorphism was associated with a QT-interval prolongation in male but not female type 1 long QT syndrome patients harbouring the *KCNQ1* Gly589Asp mutation. *KCNE1* Asp85Asn may thus be a sex-specific QT-interval modifier in type 1 LQTS. Similarly, the Ile148Met (rs738409) *PNPLA3* polymorphism is a disease modifier in primary sclerosing cholangitis with bile duct stenosis, but only in male patients (Friedrich et al. 2013). Finally, a Val89Leu polymorphism (rs523349) in the steroid 5α-reductase type 2 (*SRD5A2*) gene, which serves to reduce the conversion of testosterone to dihydrotestosterone, has been claimed to influence the severity of post-traumatic stress symptoms but in a male-specific fashion (Gillespie et al. 2013).

An intriguing parent-of-origin effect has been noted in two apparently unrelated retinoblastoma families with a heterozygous, low-penetrance splice site mutation (c.607+1G>T) in the *RB1* gene which causes skipping of exon 6 (Klutz et al. 2002). The abundance of the resulting nonsense (frameshifted) *RB1* mRNA relative to the wild-type was found to vary between members of one and the same family. Those individuals in family #1 who inherited the mutant *RB1* allele from their mother displayed a similar level of nonsense and wild-type *RB1* transcripts, and only one of eight carriers developed retinoblastoma. By contrast, those individuals in family #2 who inherited the mutant *RB1* allele from their fathers displayed a reduced abundance of the nonsense transcript with six of eight carriers developing retinoblastoma, indicating that the mutant transcript has residual function. Assuming that this is not a chance result (Fisher’s exact test; *p* = 0.04), it may be that the gender of the transmitting parent can influence the penetrance of the pathogenic mutation.

There is good evidence to suggest that sex-specific genomic architecture can influence the expression of human phenotypes, including disease traits (Ober et al. 2008). It is likely that the underlying mechanism is differential gene regulation in males and females, particularly in relation to sex steroid-responsive genes (Zhang et al. 2007; Dimas et al. 2012).

Another mechanism by which sex influences penetrance is via genomic imprinting. Genomic imprinting results from the epigenetic modification of a gene or gene region that leads to the mutually exclusive expression of either the maternal or the paternal allele. Imprinted alleles are silenced (by DNA methylation or histone modification), so that the corresponding genes are expressed only from the non-imprinted allele inherited from the other parent. In the case of disease genes, imprinting can influence the penetrance of pathological mutations depending upon whether the wild-type or the mutant allele is imprinted. Genomic imprinting can give rise to markedly different levels of clinical penetrance depending upon the parental origin of the disease allele. Examples include *SGCE* mutations in myoclonus dystonia (Zimprich et al. 2001; Müller et al. 2002; Grabowski et al. 2003) and *SDHD* mutations in paraganglioma (Badenhop et al. 2001; Simi et al. 2005; Baysal et al. 2011). In both cases, maternal imprinting ensures that the pathologically effective mutations are almost invariably inherited from the father. Intriguingly, in one family with pseudohypoparathyroidism type 1b, Jan de Beur et al. (2003) reported a case of the incomplete penetrance of an imprinting mutation. These authors found that both the clinically affected and unaffected siblings had inherited the same *GNAS1* allele from their affected mother, indicating that some dissociation must have occurred between the genetic *GNAS1* defect responsible for the disease and its epigenetic mark. The inconsistent acquisition of a paternal epigenotype on a maternal *GNAS1* allele would appear to provide evidence for the incomplete expression of a reprogramming defect that affects imprinting.

## Age-dependent penetrance

Age-dependent penetrance is present if the clinical symptoms of a given disease are increasingly likely to manifest themselves with increasing age of the at-risk individual. Age-dependent penetrance has been reported for mutations in a wide variety of different human disease genes, e.g. *MYBPC3* in hypertrophic cardiomyopathy (Michels et al. 2009; Page et al. 2012), *LMNA* in Emery–Dreifuss muscular dystrophy (Vytopil et al. 2002), *MC4R* in familial obesity due to melanocortin-4 receptor deficiency (Stutzmann et al. 2008), *GBA* in Parkinson disease (Anheim et al. 2012; Rana et al. 2013), *BRCA1* and *BRCA2* in breast cancer susceptibility (Chen and Parmigiani 2007; Al-Mulla et al. 2009; Mavaddat et al. 2013), *MEN1* in multiple endocrine neoplasia type 1 (Machens et al. 2007), *RET* in multiple endocrine neoplasia type 2A (Frank-Raue et al. 2011) and *SDHD* and *SDHB* in predisposition to paragangliomas (Hensen et al. 2010; Hes et al. 2010). The *APOE* ε4 allele (comprising the T allele of rs429358 and the C allele of rs7412 in *cis*) serves to reduce the age of onset of Alzheimer disease from 78.4 years in patients lacking the allele, to 75.3 in heterozygous carriers to 72.9 in carriers of two *APOE* ε4 alleles (Sando et al. 2008).

Age-dependent penetrance is particularly evident where large numbers of heterozygous carriers harbouring specific gene mutations have been identified by cascade screening, e.g. *LRRK2* Gly2019Ser (rs34637584) in Parkinson disease (Latourelle et al. 2008; Healy et al. 2008; Sierra et al. 2011), *GLUT1* Arg232Cys in familial idiopathic generalized epilepsy (Striano et al. 2012), *RET* Cys634Trp (rs77709286) in multiple endocrine neoplasia type 2A (Milos et al. 2008), *ACADM* Lys329Glu (rs77931234) in medium-chain acyl-CoA dehydrogenase deficiency (Andresen et al. 2012), *PKP2* Gln59Leu in arrhythmogenic right ventricular cardiomyopathy (Lahtinen et al. 2008) and *MYBPC3* c.2308+1G>A (rs112738974) in hypertrophic cardiomyopathy (Oliva-Sandoval et al. 2010). However, there are always anomalous cases; thus, in a family segregating a pathogenic missense mutation (Arg1205His) in the vacuolar protein sorting 35 (*VPS35*) gene, six family members between the ages of 54 and 73 years exhibited signs of Parkinson disease, but one individual was still asymptomatic at age 86 (Nuytemans et al. 2013).

Specific mutations may sometimes differ from each other in terms of the average age of onset of clinical symptoms. Thus, for example, patients with maturity-onset diabetes of the young (MODY) who harbour mutations in exons 9 or 10 of the *HNF4A* gene have been found to develop disease much later (average 40 vs. 24 years) than MODY patients with mutations in exons 2-8 (Harries et al. 2008). This difference in age-related penetrance is thought to be a consequence of the exon 9 and 10 mutations being absent from three of the nine HNF4A isoforms encoded by the *HNF4A* gene, whereas the mutations located in exons 2–8 affect all nine isoforms.

In some cases, the clinical penetrance of a particular mutation can change quite dramatically with age. For example, the cumulative incidence among carriers of the Arg1441Gly mutation in the *LRRK2* gene causing Parkinson disease was found to be 12.5 % until the age of 65 years, but 83 % until age 80 (Ruiz-Martínez et al. 2010). However, the penetrance of the common *TTR* Val30Met mutation causing autosomal dominant familial amyloid polyneuropathy has been estimated to be 1.7 % until the age of 30 years, 22 % until the age of 60, but still only 69 % until age 90 (Hellman et al. 2008). Majounie et al. (2011) showed that the pathogenic GGGGCC hexanucleotide expansion in the *C9orf72* gene associated with a high proportion of cases of amyotrophic lateral sclerosis and frontotemporal dementia was non-penetrant in individuals younger than 35 years, 50 % penetrant by age 58 but almost fully penetrant by age 80. Age-dependent penetrance could thus provide another explanation for why some putatively pathological mutations listed in HGMD are present in apparently healthy individuals from the 1000 Genomes Project.

A glimpse of the way ahead is provided by a recent study of symptomatic and asymptomatic carriers of a specific granulin (*GRN*) mutation (Thr272Ser) responsible for autosomal dominant frontotemporal lobar degeneration (FTLD), a disease whose onset typically occurs in the sixth decade of life (Milanesi et al. 2013). Unsurprisingly, both the symptomatic and asymptomatic *GRN* mutation carriers had lower serum levels of progranulin than non-carriers. However, using whole-genome microarray analysis, the leukocyte expression of the *TMEM40* and *LY6G6F* genes was found to be significantly higher in FTLD patients harbouring *GRN* mutations as compared to asymptomatic carriers. Further, elevated expression of the genes was correlated with increased brain damage and could therefore be directly related to the pathology of the disease (Milanesi et al. 2013).

## Epigenetic influences on disease penetrance

As briefly discussed above in the context of the influence of gender upon penetrance, epigenetic modifications may also account for incomplete penetrance. Thus, when monozygotic twins are discordant for disease phenotypes, epigenetic differences should be considered (Wong et al. 2005; Kaminsky et al. 2009; Gordon et al. 2011; 2012). Indeed, monozygotic twins have been reported who differ both in relation to a specific clinical phenotype and in terms of an epigenotype. For example, monozygotic twins discordant for childhood leukaemia have been found to have discordant *BRCA1* methylation status (Galetzka et al. 2012). Similarly, hypermethylation of *SLC6A4*, encoding the serotonin transporter, has been reported in one member of a monozygotic twin pair discordant for bipolar disorder (Sugawara et al. 2011).

Epigenetic differences may also contribute to incomplete penetrance in other conditions such as asthma, where DNA methylation has been reported to modulate the risk of disease conferred by genetic variants at the zona pellucida binding protein 2 (*ZPBP2*; Berlivet et al. 2012), forkhead box P3 (*FOXP3*; Runyon et al. 2012), interferon-γ (*IFNG*; Runyon et al. 2012) and interleukin-4 receptor (*IL4R*; Soto-Ramírez et al. 2013) gene loci.

A special case of imprinting is provided by X-inactivation (Dobyns et al. 2004). When a disease gene is X-linked, skewed X-inactivation can cause variable penetrance of pathogenic mutations in female carriers (Van den Veyver 2001). Examples of this phenomenon involve mutations in the *TIMM8A* gene (Xq22.1) in dystonia-deafness syndrome (Plenge et al. 1999), the *EBP* gene (Xp11.23) in X-linked dominant chondrodysplasia punctata (Shirahama et al. 2003), the *FLNA* gene (Xq28) in otopalatodigital type 1 syndrome (Hidalgo-Bravo et al. 2005), the *ABCD1* gene (Xq28) in a family with X-linked adrenoleukodystrophy (Wang et al. 2013b) and the *ZIC3* gene (Xq26.3) in a family with complex heart defects (Chhin et al. 2007). It should, however, be pointed out that some *ZIC3* mutations are characterized by reduced penetrance in males, a finding that cannot be explained by skewed X-inactivation (Mégarbané et al. 2000).

## Gene–environment interactions and penetrance

The environment, in its broadest sense, will often influence clinical penetrance, either ameliorating or exacerbating the impact of heritable genetic variants (Hunter 2005). Indeed, environmental modifiers of disease penetrance (e.g. diet, alcohol intake, drugs, metabolic syndrome) have long been known to influence the penetrance of *HFE* C282Y homozygosity in haemochromatosis (Beutler 2003; Rossi et al. 2008; Deugnier and Mosser 2008).

One way to explore the relative contribution of genes and environment is by studying monozygotic twins harbouring the same pathogenic mutation(s) and sharing the same genetic background. Although the vast majority of such monozygotic twin pairs have been found to be concordant in terms of their clinical phenotypes (e.g. Miesfeldt et al. 1998; Munhoz et al. 2008; McDade et al. 2012), others are quite discordant (Matsuo et al. 2000; Amann et al. 2001; Martin et al. 2003; Holmgren et al. 2004; Lachmann et al. 2004; Czlonkowska et al. 2009; Biegstraaten et al. 2011; Fencl et al. 2012; Iatropoulos et al. 2012), suggesting that the environment can often play an important role in determining both the penetrance and expressivity of pathological mutations. [It should be borne in mind that there are various alternative genetic explanations for discordant phenotypes in monozygotic twins, including de novo post-zygotic mutation (Kentsis et al. 2009; Vogt et al. 2011), compensatory mutations (Mankad et al. 2006) and somatic copy number variation (Bruder et al. 2008) that obviate the need for a major contribution from the environment, as well as acquired epigenetic differences (Galetzka et al. 2012; Bennett et al. 2008)].

An environmental influence on penetrance is perhaps at its most evident in cancer susceptibility (Houlston and Peto 2004; Shen 2009). Indeed, an environmental component is very important in colorectal cancer where inherited genetic variants at a number of different loci interact primarily with dietary variables and overweight to confer risk (Hutter et al. 2012; Siegert et al. 2013). In similar vein, inherited differences in skin pigmentation influence the risk of melanoma, but this risk is further modified both by latitude of habitation and lifestyle choices (van der Velden et al. 2001; Bishop et al. 2002; Begg et al. 2005; Meyle and Guldberg 2009; Scherer and Kumar 2010). Parity and breast feeding are both known to be modifiers of risk of breast/ovarian cancer in *BRCA1* mutation carriers (McLaughlin et al. 2007; Jernström et al. 2004; Cullinane et al. 2005; Antoniou et al. 2006). Another example of a gene–environment interaction in the context of cancer is provided by cytochrome P450 gene variants that may influence cancer risk by virtue of their roles in xenobiotic metabolism, detoxification of carcinogens, and to a lesser extent the bioactivation of procarcinogens (Rodriguez-Antona et al. 2010). Lung cancer provides an excellent example of the interaction of genes and environment. Three different GWAS, published virtually simultaneously, provided the first convincing evidence for an association between heritable genetic variation at the nicotinic acetylcholine receptor *CHRNA5*/*CHRNA3*/*CHRNB4* locus on chromosome 15q25.1 and lung cancer (Amos et al. 2008; Hung et al. 2008; Thorgeirsson et al. 2008). Although allele T of SNP rs1051730, a synonymous variant located within exon 5 of the *CHRNA3* gene, was found to be strongly associated with smoking quantity, the issue of whether the association with lung cancer was direct or indirect (i.e. mediated through cigarette smoking and nicotine dependence) remained unclear. Galvan and Dragan (2009) performed a meta-analysis of reported studies of the 15q25 region and found that this locus was not associated with lung cancer risk in never-smokers. This lack of effect argued for an indirect effect of genetic variation at the 15q25 locus on lung cancer risk via an association between these variants and smoking/nicotine dependence. However, Wang et al. (2010b) subsequently examined the relationship between rs1051730 and lung cancer and concluded that, in addition to its indirect influence on disease risk (through smoking behaviour), this variant also exerted a rather larger (and direct) effect. Kaur-Knudsen et al. (2011) concurred, demonstrating that homozygosity for rs1051730 was associated with a smoking behaviour-adjusted relative risk of lung cancer of 1.6, indicating that rs1051730 is associated with an additional risk of lung cancer over and above that derived from its effect on smoking behaviour. Finally, in a lung cancer case–control study, VanderWeele et al. (2012) employed two 15q25.1 SNPs, rs8034191 and rs1051730, to show that the proportion of increased risk due to smoking was only 3.2 % and that the association of the 15q25 variants with lung cancer operates primarily through pathways other than smoking behaviour. All of the above notwithstanding, the risk of lung cancer conferred directly or indirectly by genetic variants on 15q25 would be small if the individual concerned simply opted not to smoke (Brennan et al. 2011).

The penetrance of genetic variants conferring susceptibility to infectious disease is clearly contingent upon exposure to the specific pathogens concerned (Vannberg et al. 2011; Chapman and Hill 2012). One example is the *CCR5* 32-bp (c.554del32) deletion which is associated with a lower rate of HIV infection and a delay in the onset of AIDS (Smith et al. 1997). Sex may also play a role in some conditions; thus, in multiple sclerosis, women appear to be more responsive to the environmental risk factors that cause the disease (Goodin 2012; O’Gorman et al. 2012).

Diet is also an important modifier of clinical penetrance. Thus, an inherited predisposition to obesity (exemplified by the association between dietary fat intake and obesity in carriers of the *PPARG2* Pro12Ala allele; Memisoglu et al. 2003) is in principle modifiable by diet (Walters et al. 2010; Ramachandrappa and Farooqi 2011). Similarly, the impact of genetic variants at the *FTO* locus on risk of obesity can be attenuated by physical activity (Kilpeläinen et al. 2011). Diet is also an important modifier of clinical penetrance in phenylketonuria, as mentioned in the “Introduction” to this review, where the penetrance of the condition can be very substantially reduced by restricting dietary phenylalanine (van Spronsen 2010).

Heavy coffee drinkers have been known for some time to have a reduced risk of developing Parkinson disease. However, the risk of developing Parkinson disease has been found to be reduced even further for heavy coffee drinkers by a specific variant in the *GRIN2A* gene; compared to light coffee drinkers with an rs4998386_CC genotype, heavy coffee drinkers with the same genotype have an 18 % lower risk, whereas heavy coffee drinkers with an rs4998386_TC genotype have a 59 % lower risk (Hamza et al. 2011).

More unusually, altitude has been reported to act as a modifier of the phenotypic severity of hereditary paraganglioma type 1 caused by mutations in the succinate dehydrogenase D (*SDHD*) gene (Astrom et al. 2003). Since chronic hypoxic stimulation at high altitude causes sporadic carotid body paragangliomas, Astrom et al. (2003) proposed that SDHD might be involved in oxygen sensing. Thus, whilst *SDHD* mutations could impair oxygen sensing, low altitude may serve to reduce the penetrance of these mutations.

The clinical penetrance of psychological disorders and traits has long been known to be strongly influenced by gene–environment interactions (Dick 2011). For example, a 44-bp deletion/insertion polymorphism in the promoter region of the serotonin transporter gene *SLC6A4* was reported to be associated with depression after stressful life events (Caspi et al. 2003). Recently, Klengel et al. (2013) gave us a glimpse of the likely complexity of the mechanisms underlying gene–environment interactions in the context of psychological disorders. These authors demonstrated that a risk variant for post-traumatic stress disorder and major depression in the FK506-binding protein 5 gene (*FKBP5*) is demethylated in several cell types in children exposed to trauma. This demethylation persists into adulthood and confers an increased risk of developing disease. In *FKBP5* risk allele carriers, excessive cortisol release during early life stress leads to demethylation within the glucocorticoid-responsive elements of *FKBP5* in intron 7 with the consequence of long-lasting disruption of the ultra-short feedback loop that balances FKBP5 and glucocorticoid receptor activity, causing dysregulation of the stress hormone system. The *FKBP5* risk allele corresponds to a functional polymorphism located in intron 2 of the *FKBP5* gene that alters the chromatin interaction between the transcriptional start site and long-range enhancers, thereby increasing the transcriptional activity of *FKBP5* over and above that of the wild-type allele. Only the risk allele is able to form a three-dimensional complex which includes RNA polymerase II and a glucocorticoid-responsive element located within intron 7 of *FKBP5*. Enhanced transcription of the risk allele facilitates the PolII-dependent demethylation in intron 7 in response to elevated glucocorticoids under early life stressful conditions. The reduced methylation of intron 7 CpGs leads to increased induction of *FKBP5* by glucocorticoid receptor activation, especially in risk allele carriers, representing an enhancement of the ultra-short feedback loop leading to increased glucocorticoid receptor resistance. If this occurs during developmentally critical periods, then the methylation patterns will remain stable over time (Klengel et al. 2013). Hence, the demethylation in *FKBP5* intron 7 depends upon both childhood trauma and the sequence variant in intron 2, in a tripartite gene–environment interaction (Szyf 2012).

The above examples represent the tip of the iceberg because the clinical penetrance of most monogenic conditions and all complex disease is likely to be influenced by the environment in some way or another. A further glimpse of this complexity is perhaps provided by Kallberg et al. (2007) who reported interactions between *HLA*-*DRB1* SE alleles, the A allele of the *PTPN22* Arg620Trp (rs2476601) polymorphism and smoking in conferring risk of rheumatoid arthritis. Gene–environment interactions are also evident in asthma (Custovic et al. 2012; Chang et al. 2012) and eczema (Bisgaard et al. 2008) and will become increasingly apparent in other disorders as new techniques are developed to identify them (Aschard et al. 2012). To this end, mouse models are beginning to come into their own as a means to study the role of gene–environment interactions in the aetiology of human disorders; by these means, short-term gestational hypoxia has been found to increase the penetrance of vertebral defects in congenital scoliosis (Sparrow et al. 2012).

## Conclusions

**Fig. 1:**
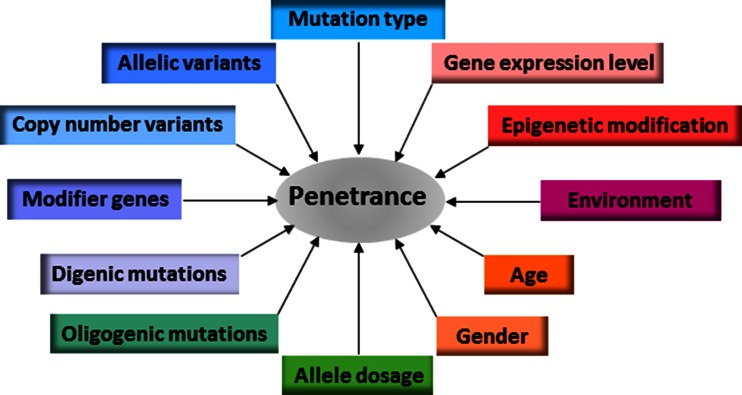
Some of the different mechanisms underlying the phenomenon of reduced penetrance in human inherited disease

A holy grail of human medical genetics is to be able to deduce the likely clinical phenotype of an individual from their genotype or genomic sequence. It was once perhaps naively assumed that, at least for “monogenic” disorders, genotype–phenotype relationships would be that simple, and also fairly straightforward to discern. However, it has been clear for some time that it is inappropriate to regard such disorders as either simple or monogenic in any strict sense. Further, in many cases, the reality is that we cannot readily draw straight lines of causation from known genotypes to specific clinical phenotypes. This is because instances abound of individuals who harbour a disease-associated mutation/genotype, but who do not express certain features of the disease or who may even be asymptomatic. This phenomenon of reduced penetrance may or may not be the norm, but it is far from being a rare exception. Our appreciation of its full extent is still emerging, although some of the different mechanisms underlying reduced penetrance are now becoming apparent (Fig. 1).

It has become clear from large-scale sequencing studies that many individuals in the general population harbour large numbers of potentially disadvantageous variants without suffering any obvious ill effects (The 1000 Genomes Project Consortium 2010; MacArthur et al. 2012; Xue et al. 2012; Shen et al. 2013a). Thus, it would appear that many mutations are, on their own, insufficient to cause disease and need to occur in the presence of other genetic variants, either allelic or non-allelic, as well as facultative environmental factor(s), for a disease state to ensue. Indeed, many pathological mutations may only be conditionally pathogenic, exerting a detrimental effect only if and when the genetic and external environments interact to push the phenotype over some notional threshold into pathology.

Penetrance is best thought of as being a genotype-specific rather than a gene-specific or disease-specific phenomenon. Thus, in any given disease gene, some mutations may exhibit complete penetrance, whereas others may show incomplete or even quite low penetrance. Generally speaking, mutations that display low penetrance also tend to exert milder effects on the clinical phenotype and/or protein function, while the more highly penetrant a mutation is, the less frequent it is likely to be in the general population (Coventry et al. 2010; Marth et al. 2011; Gorlov et al. 2011; Tennessen et al. 2012; Nelson et al. 2012; Subramanian 2012; Fu et al. 2013). Whereas highly penetrant mutations may exert their pathogenic effects with relatively little interaction with other genetic or environmental factors, low-penetrance mutations are generally characterized by significant gene–gene and gene–environment interactions (Cordell 2009). Different combinations of such variants may contribute to the variable penetrance characteristic of both monogenic and complex disease.

Whatever the molecular basis may be in the case of a given mutation, reduced penetrance is in general likely to present a serious impediment to the implementation of any scheme designed to classify the pathological significance of human genetic variants (e.g. Plon et al. 2008; Tavtigian et al. 2008). Reduced penetrance is also likely to present problems in identifying pathological mutations in whole-genome/exome sequencing programmes unless it is explicitly built into the disease models being considered (Varga et al. 2013). Despite the complexities it introduces, it also offers hope in the sense that if we are able to identify environmental factors, drugs or other types of intervention that serve to reduce the penetrance of a given pathological variant, or alternatively delay the onset of its pathological sequelae beyond the natural lifespan of its carrier, we shall have a whole new range of therapeutic approaches at our disposal.

Human genetic variation occurs as a continuum ranging from neutral polymorphisms, through functional polymorphisms and disease susceptibility variants to true pathological mutations with high penetrance. However, in addition and as discussed above, it has become increasingly clear that our genomes contain many ‘putatively disadvantageous variants’ that are probably insufficient on their own to cause disease, but nevertheless still have the potential to contribute to pathogenesis. Since it has also become clear that many genetic disorders are not monogenic as originally supposed, but may instead involve mutations in two or more genes, we speculate that different combinations of pathological mutations with low penetrance, functional polymorphisms, disease susceptibility variants and ‘putatively disadvantageous variants’ may vary quite considerably in terms of their net functional and hence clinical effect. Such combinations are likely to exert an influence on the age of onset and/or clinical severity of the disease in question.

The rationale of genetic studies of complex phenotypes has generally relied upon either the ‘common disease, common variant (CDCV)’ hypothesis or the ‘common disease, rare variant (CDRV)’ hypothesis. The former postulates that complex phenotypes result from the cumulative effects of a number of common variants, each with a modest effect size and relatively low penetrance. The latter proposes that complex phenotypes result from multiple rare variants, each with relatively high penetrance and large effect sizes (Schork et al. 2009). It is highly likely that both rare and common alleles will contribute to complex phenotypes and so effect sizes may be expected to differ quite widely, with rare variants with large effects complemented by a large number of frequent variants with small effects. The clinical phenotypes of complex phenotypes are therefore likely to be due to individual effects of, and interaction between, multiple causative or contributory alleles, as well as non-genetic determinants.

The full relevance of digenic and oligogenic inheritance to the phenomenon of incomplete or variable penetrance remains to be elucidated. If, however, it turns out that digenic and oligogenic conditions are more common than originally anticipated, then many disease contributory variants will have evaded purifying selection, and hence those variants that in combination (but not individually) have significant pathogenic potential will not be as infrequent as might be expected under the CDRV hypothesis. Since both multiple common and rare variants may be involved in conferring disease susceptibility, we are not obliged to favour either the CDCV hypothesis or the CDRV hypothesis. Moreover, in view of the likely complexity of the gene–gene interactions involved, we concur with Lupski et al. (2011) that “for a given individual, what is important to know is not only the number and location of pathogenic variants taken one at a time, but also the unique composition of his or her genome-wide mutational burden”.

Lupski et al. (2011) charted progress on the road to a unified genetic model for human disease and opined that such a model should unite categories of diseases, previously held to be distinct entities, as part of a continuum which would include chromosomal syndromes, genomic disorders, Mendelian traits and common diseases or complex traits. Concurring with this view, we envisage an integrated concept of genetic aetiology in which different types of mutation (from single base substitutions to copy number variants), different combinations of mutations in multiple genes (whether in homozygosity or heterozygosity), *cis*-acting or *trans*-acting modifiers, common variants, rare variants, de novo variants and even somatic variants, jointly serve to exacerbate or ameliorate a given clinical phenotype. Further, to explain the scale of reduced penetrance, we need to conceptualize clinical phenotypes as being derived, potentially at least, from the expression of different genetic variants in two or more genes. On the basis of the data collated for this review, it seems reasonable to conclude that digenic, oligogenic and polygenic influences are much more frequent than has perhaps hitherto been realized. Unravelling such influences will undoubtedly be key to understanding the molecular basis of reduced penetrance. The impact of disease genotypes may also be modified by epigenetic and environmental factors, allowing both for synergistic and antagonistic interactions resulting in highly individualized contributions to the phenotype (whether deleterious or protective) that will variously perturb the balance of specific biological pathways so as to give rise to disease.

With the advent of next-generation sequencing, very large numbers of genetic variants are being detected in individual genomes and it has been necessary to develop new algorithms to identify those variants which are of key functional/clinical importance. However, if in using these tools, we focus exclusively on single infrequent variants under the assumption that they will invariably exert their effects in splendid isolation, then there is a very real danger that we shall inadvertently exclude from consideration those more frequent variants with modest effects, blithely ignoring their potential for interaction with the rare variants. The irony would then be that, despite having the requisite mutation and polymorphism data available, the molecular basis of genotype–phenotype relationships in many inherited diseases (including, of course, the phenomenon of reduced penetrance) could still remain unintelligible. The alternative, anticipating multigenic influences on the clinical phenotypes associated with disorders traditionally regarded as being monogenic, should not only to lead to new insights into the nature of reduced penetrance, but is also likely to improve our understanding of the nature of complex disease.
